# Plastid Phylogenomic Data Offers Novel Insights Into the Taxonomic Status of the *Trichosanthes kirilowii* Complex (Cucurbitaceae) in South Korea

**DOI:** 10.3389/fpls.2021.559511

**Published:** 2021-07-27

**Authors:** Inkyu Park, Jun-Ho Song, Sungyu Yang, Sungwook Chae, Byeong Cheol Moon

**Affiliations:** ^1^Herbal Medicine Resources Research Center, Korea Institute of Oriental Medicine, Naju, South Korea; ^2^Herbal Medicine Research Division, Korea Institute of Oriental Medicine, Daejeon, South Korea

**Keywords:** *Trichosanthes*, plastid genome, divergent region, phylogenetic relationship, indel marker, herbal medicine

## Abstract

*Trichosanthes* is a genus in Cucurbitaceae comprising 90–100 species. *Trichosanthes* species are valuable as herbaceous medicinal ingredients. The fruits, seeds, and roots of species such as *T. kirilowii* and *T. rosthornii* are used in Korean traditional herbal medicines. *T. rosthornii* is only found in China, whereas in South Korea two varieties, *T. kirilowii* var. *kirilowii* and *T. kirilowii* var. *japonica*, are distributed. *T. kirilowii* var. *kirilowii* and *T. kirilowii* var. *japonica* have different fruit and leaf shapes but are recognized as belonging to the same species. Furthermore, although its members have herbal medicine applications, genomic information of the genus is still limited. The broad goals of this study were (i) to evaluate the taxonomy of *Trichosanthes* using plastid phylogenomic data and (ii) provide molecular markers specific for *T. kirilowii* var. *kirilowii* and *T. kirilowii* var. *japonica*, as these have differences in their pharmacological effectiveness and thus should not be confused and adulterated. Comparison of five *Trichosanthes* plastid genomes revealed locally divergent regions, mainly within intergenic spacer regions (*trnT-UGU–trnL-UAA*: marker name Tri, *rrn4.5–rrn5*: TRr, *trnE-UUC–trnT-GGU*: TRtt). Using these three markers as DNA-barcodes for important herbal medicine species in *Trichosanthes*, the identity of *Trichosanthes* material in commercial medicinal products in South Korea could be successfully determined. Phylogenetic analysis of the five *Trichosanthes* species revealed that the species are clustered within tribe Sicyoeae. *T. kirilowii* var. *kirilowii* and *T. rosthornii* formed a clade with *T. kirilowii* var. *japonica* as their sister group. As *T. kirilowii* in its current circumscription is paraphyletic and as the two varieties can be readily distinguished morphologically (e.g., in leaf shape), *T. kirilowii* var. *japonica* should be treated (again) as an independent species, *T. japonica*.

## Introduction

*Trichosanthes* L. is a large genus of the tribe Sicyoeae Schrad. (Cucurbitaceae Juss.) comprising 90–100 species (de Wilde and Duyfjes, 2010; de Boer and Thulin, 2012). The members of the genus are diecious, rarely monecious, perennial climbing herbs, characterized by unlobed or palmately lobed, rarely compound leaves with branched tendrils, a usually distinct fimbriate corolla, and ovoid to globose or elongated fusiform pepos (Huang et al., 2011; de Boer and Thulin, 2012). The center of diversity for the genus is Southeast Asia, and its range is India to East Asia and southeast Australia (de Wilde and Duyfjes, 2010; Huang et al., 2011; de Boer and Thulin, 2012). *Trichosanthes* is of broader interest for several reasons, including being a model plant for sex determination in plants and for its medicinal properties (de Boer et al., 2012; Yu et al., 2018; Lu et al., 2021). South Korea hosts two varieties of a *Trichosanthes* species: *T. kirilowii* var. *kirilowii* and *T. kirilowii* var. *japonica* (Kim and Choi, 2018). *T. kirilowii* var. *japonica* is distinguished from *T. kirilowii* var. *kirilowii* based on the color of its fruits. In addition, the two *T. kirilowii* varieties are distinguished based on leaf shape as well as seed color (Ohba, 1999).

Sequence data from the plastid genome has made it possible to identify medicinal plants (Park et al., 2018b, 2019a,b, 2020). *T. kirilowii* and *T. rosthornii* are important in Korean and Chinese traditional herbal medicine, as their roots and seeds are used as Trichosanthis Radix and Trichosanthis Semen (herbal medicinal names), respectively. Such medicines are used for their immunomodulatory, anti-tumor, and anti-HIV properties (Yu et al., 2018). Furthermore, *T. kirilowii* and *T. rosthornii* fruits are used in traditional Chinese medicine as Trichosanthis Fructus and Trichosanthis Pericarpium, respectively. *T. kirilowii* var. *japonica* has been reported to prevent proliferation of leukemia cell lines *in vitro* (Kim et al., 2003). For quality control and to ensure the safety and effectiveness of their ingredients, only the Korean Ministry of Food and Drug Safety designates and regulates Trichosanthis Radix and Trichosanthis Semen and the roots and seeds of *T. kirilowii* and *T. rosthornii* as medicine (Korea Institute of Oriental Medicine [KIOM], 2020). Since *T. kirilowii* var. *japonica* is not regulated by law, the use of *T. kirilowii* var. *japonica* as a medicine in South Korea poses a problem. There are some efficacy reports, but there are insufficient reports related to pharmacological studies, component analysis, or physicochemical composition. Trichosanthis Radix and Trichosanthis Semen are obtained in the form of slices and powders in Korean traditional markets. In general, distinguishing authentic from inauthentic herbal products is challenging for the untrained eye. Therefore, appropriate methods are required to discriminate good quality herbal products from adulterated preparations. Adulterants may cause negative side effects and quality problems. The purity of herbal medicine ingredients can be tested using species-specific molecular markers. As mentioned above, *T. kirilowii* var. *kirilowii* and *T. kirilowii* var. *japonica* are distributed in South Korea. Owing to a lack of major morphological differences in their floral characteristics and their sympatric distribution in South Korea, distinguishing them is often challenging. To protect the original herbal medicine–*T. kirilowii* var. *kirilowii* and *T. rosthornii*–from adulterants, molecular markers could be used for testing and identification.

Plastid genomes are useful for species classification, identification, population genetics studies, diversity and evolutionary analysis, and can infer well-resolved phylogenetic relationships, even at the species level (Jansen et al., 2007; Parks et al., 2009). Furthermore, plastid genomes are useful for DNA barcoding approaches. Insertion/deletion (indel) genetic variants from the plastid genome are useful markers for species identification and discrimination (Dong et al., 2012; Li et al., 2015; Park et al., 2019b). Several studies have developed indel markers to identify the correct herbal medicinal plants among related species. Kim et al. (2015) reported the plastid genome of an important herbal medicinal plant, *Panax ginseng*, and indel markers from sequence variants of plastid genomes that could discriminate 14 *Panax ginseng* accessions. *Aconitum* species, which have toxic components such as aconitine, are extensively used in herbal medicine (Park et al., 2017). The complete plastid genomes of *Aconitum coreanum* and *A. carmichaelii* have revealed indel sequence variation among the *Aconitum* species. An *A. coreanum* species*-*specific marker was developed and fully distinguished nine other *Aconitum* accessions. Furthermore, indel markers were developed to distinguish the important herbal medicine Pharbitidis Semen (seeds of *Ipomoea nil* and *I. purpurea*) from closely related *Ipomoea* species (Park et al., 2018b). Such cases involving traditional herbal medicine attest to the value of plastid genomes. However, chloroplast capture, which refers to the introgression of one plastid genome into another species due to hybridization, has also been reported (Acosta and Premoli, 2010; Stegemann et al., 2012; Kawabe et al., 2018). Therefore, it is recommended that both nuclear and plastid DNA be used together for accurate species identification.

In this study, our aim was to explore the taxonomic identities of *T. kirilowii* var. *kirilowii* and *T. kirilowii* var. *japonica* and to facilitate their distinction. We also aimed to authenticate the use of *Trichosanthes* in herbal medicine. To this end, the plastid genomes of five *Trichosanthes* accessions were sequenced and compared based on nucleotide variation and genome structure. We used the data to test the phylogenetic relationships among five *Trichosanthes* taxa (four species, one with two varieties). Finally, to authenticate *Trichosanthes* species-based herbal medicine, we developed and tested novel molecular marker sets based on genetically variable plastid regions.

## Materials and Methods

### Plant Materials and Morphological Analysis

Fresh *T. kirilowii* var. *kirilowii* and *T. kirilowii* var. *japonica* leaves were collected from five natural habitats in South Korea (Supplementary Figure 1), and one *T. rosthornii* individual was collected from its natural habitat in China (Supplementary Table 1). All specimens were registered with the Korean Herbarium of Standard Herbal Resources (Index Herbariorum Code KIOM) at the Korea Institute of Oriental Medicine (KIOM). Two accessions each of *T. kirilowii* var. *kirilowii* and *T. kirilowii* var. *japonica* and one accession of *T. rosthornii* were also used for plastid genome analysis (Supplementary Table 1).

To investigate the morphology of *T. kirilowii* var. *kirilowii* and *T. kirilowii* var. *japonica*, mature leaves, fruits, and seeds from all 25 samples of each variety (five individuals × five collection sites) were selected (Supplementary Table 1). The general shape, degree of division, and hairiness were observed in detail under a stereomicroscope (Olympus SZX16, Olympus, Tokyo, Japan). The Royal Horticultural Society Color Chart^®^ (Royal Horticultural Society, 5th edition) was used to determine the color of the seeds.

### Genome Sequencing and Assembly

DNA was extracted using a DNeasy Plant Maxi Kit (Qiagen, Valencia, CA, United States) according to the manufacturer’s instructions. Illumina short-insert paired-end sequencing libraries (TruSeq DNA Nano kit) were constructed and sequenced using the NextSeq500 platform (Illumina, San Diego, CA, United States). *De novo* assembly was used to construct plastid genomes from the resulting whole-genome shotgun sequencing reads. CLC quality trim v 4.2.1 (CLC Inc., Aarhus, Denmark) was used to trim and check the quality of the reads. Trimmed paired-end reads (Phred score ≥ 20) were assembled using the CLC genome assembler v 4.2.1 (CLC Inc.) using default parameters. Principal contigs representing the plastid genome were retrieved from the total contigs using Nucmer (Delcher et al., 2003), and aligned contigs were ordered using the plastid genome sequence of *Hodgsonia macrocarpa* (NC_039628) as a reference (Zeng et al., 2018). The representative plastid contigs were arranged in order based on a previously reported plastid genome sequence and connected into a single draft sequence by joining the overlapping terminal sequences. Assembly errors were identified in the initial assembly contigs and manually corrected by the mapping of raw reads to assembled sequences. SOAP *de novo* gap closer v 0.99 was used to fill gaps based on the alignments of paired-end reads (Luo et al., 2012). LSC/IR, IR/SSC, SSC/IR, and IR/LSC regions of completed plastid genomes were validated using PCR-based sequencing. IR boundaries were amplified using 20 ng of genomic DNA in a 20-μL PCR mixture (SolgTM 2X Taq PCR smart mix 1, Solgent, Daegeon, South Korea) with 10 pmol of each primer (Bioneer, Daejeon, South Korea). Amplification was performed using a Pro Flex PCR system (Applied Biosystems, Waltham, MA, United States) according to the following program: initial denaturation at 95°C for 2 min; 35 cycles at 95°C for 1 min, 60°C for 1 min, 72°C for 1.5 min; and final extension at 72°C for 5 min. PCR products were separated on 2% agarose gels at 150 V for 40 min. To validate IR boundary sequences, each PCR product was rescued from the agarose gel, subcloned into the pGEM-T Easy vector (Promega, Madison, WI, United States), and sequenced using a DNA sequence analyzer (ABI 3730, Applied Biosystems Inc., Foster City, CA, United States) to determine sizes and verify the sequences of amplicons. Primer information and sequence alignment results are listed in Supplementary Tables 2, 3.

### Genome Annotation and Comparative Analysis

Gene annotation of the five *Trichosanthes* plastid genomes was performed using GeSeq v. 1.76 (Tillich et al., 2017). Protein-coding sequences were manually curated and confirmed using Artemis v. 1.8 (Carver et al., 2008), then checked against the National Center for Biotechnology Information (NCBI) protein database. The tRNAs were confirmed with tRNAscan-SE 1.21 (Lowe and Eddy, 1997). IR region sequences were confirmed using IR finder and RepEx v. 1.0 (Michael et al., 2019). Circular maps of the five *Trichosanthes* plastid genomes were obtained using OGDRAW v. 1.3.1 (Greiner et al., 2019). GC content and relative synonymous codon usage (RSCU) of all five *Trichosanthes* plastid genomes were analyzed using MEGA6 (Tamura et al., 2013). The codon usage distribution of 33 Cucurbitaceae plastid genomes was visualized using the Heatmapper program utilizing an average linkage clustering method and the Euclidean distance measurement method (Babicki et al., 2016). The mVISTA program v. 2.0 was used in Shuffle-LAGAN mode to compare the five *Trichosanthes* plastid genomes with the *T. kirilowii* var. *kirilowii* 1 plastid genome as a reference. DnaSP v. 6.1 was used to calculate nucleotide variability (Pi) among the five *Trichosanthes* plastid genomes (Rozas et al., 2017). Pi value represented nucleotide variability as a measure of genetic variation at the nucleotide level for five *Trichosanthes* accessions. Each plastid genome was divided into genes, introns, and intergenic regions.

### Repeat Analysis

Tandemly arranged repeats of short DNA motifs, 1–6 bp in length, called simple sequence repeats (SSRs), were detected in the five *Trichosanthes* plastid genomes using MISA-web^1^ (Beier et al., 2017). The following criteria were used for detecting SSRs: SSR motif length between one and six nucleotides with the minimum number of repeat parameters set to 10, 5, 4, 3, 3, and 3 for mono-, di-, tri-, tetra-, penta-, and hexa-nucleotides, respectively. Tandem repeats (>20 bp) were identified using the Tandem Repeats Finder v. 4.07 (Benson, 1999) using a minimum alignment score and maximum period size of 50 and 500, respectively, and the identity of repeats was set to ≥90%.

### Indel Marker Development and *Trichosanthes* Validation

To detect species-specific variants, we aligned the five *Trichosanthes* plastid genomes using MAFFT v. 7 (Katoh et al., 2002). Three regions with indels (hereafter named Tri, TRr, and TRtt), located in the intergenic spacers *trnT-UGU–trnL-UAA* (Tri), *rrn4.5–rrn5* (TRr), and *trnE-UUC–trnT-GGU* (TRtt), were selected as candidate marker regions, and the sequences of the whole intergenic spacers were extracted from the aligned plastid genomes of all species using Bioedit v. 7.2 (Hall, 1999). Primers flanking the three variable indel regions were designed using Primer-BLAST^2^. The specificity of the indel markers was confirmed using PCR amplification with 20 ng of genomic DNA extracted from 23 samples of *Trichosanthes* species in a 20-μl PCR mixture (Solg^TM^ 2 × Taq PCR smart mix 1, Solgent, Daejeon, South Korea) with 10 pmol of each of the Tri, TRr, and TRtt primers. Tri, TRr, and TRtt were amplified on a Pro Flex PCR system (Applied Biosystems, Waltham, MA, United States) with the following amplification parameters: initial denaturation at 95°C for 2 min; 35 cycles at 95°C for 50 s, 62°C for 50 s, 72°C for 50 s, and a final extension at 72°C for 5 min. PCR products were separated on 2% agarose gel for 40 min at 150 V. Each PCR product was isolated using a gel extraction kit (Qiagen), subcloned into a pGEM-T Easy vector (Promega, Madison, WI, United States), and sequenced using a DNA sequence analyzer (ABI 3730, Applied Biosystems Inc., Foster City, CA, United States). The *Trichosanthes* accessions used are listed in Supplementary Table 4. The commercial products are listed in Supplementary Table 5. Tri, TRr, and TRtt primer sequences are listed in Supplementary Table 6.

### Phylogenetic Analysis

A total of 19 plastid genomes were used for phylogenetic analyses: 17 from *Trichosanthes* species and one each from *Cucumis melo* var. *makuwa* (MF536700) and *Cucumis melo* var. *momordica* (MF536701) as the outgroup. Of these, 12 plastid genome sequences were downloaded from NCBI GenBank (Supplementary Table 7). MAFFT (Katoh et al., 2002) was used to align the plastid genomes, and alignments were manually adjusted using Bioedit (Hall, 1999). Subsequently, each aligned gene (CDS) was extracted using Geneious prime v. 2021.1^3^, yielding 58 single CDS alignments. The alignment files were filtered to remove ambiguously aligned regions using GBLOCKS v. 0.91b (Castresana, 2000), and concatenated using Geneious. The best-fitting model of nucleotide substitutions was determined based on Akaike Information Criterion in JModeltest v. 2.1.10 (Darriba et al., 2012). Maximum likelihood (ML) analysis was performed using RaxML v. 8.0.5 (Stamatakis, 2014) with 1,000 bootstrap replicates based on the GTR + I + G model. Bayesian Inference (BI) analysis was carried out using MrBayes v. 3.2.2 (Ronquist et al., 2012), with two independent runs and four chains run simultaneously for 5,000,000 generations. Trees were sampled every 100,000 generations, with the first 25% discarded as burn-in. The 50% majority-rule consensus tree was visualized using Figtree v. 1.4.2 (Rambaut, 2014), with posterior probabilities (PP) estimated from the sampled trees after the burn-in fraction was discarded. Nuclear ITS sequences were obtained using the above method (Indel marker development and *Trichosanthes* validation) using KIOM specimens (Supplementary Table 1). The ITS region was amplified using ITS1 (TCC GTA GGT GAA CCT GCG G) and ITS4 (TCC GCT TAT TGA TAT GC) primers, as described previously (White et al., 1990). Phylogenetic analysis was carried out using the method applied for the plastid genomes.

## Results

### Morphological Characteristics of *Trichosanthes kirilowii* vars. *kirilowii* and *japonica*

**FIGURE 1:**
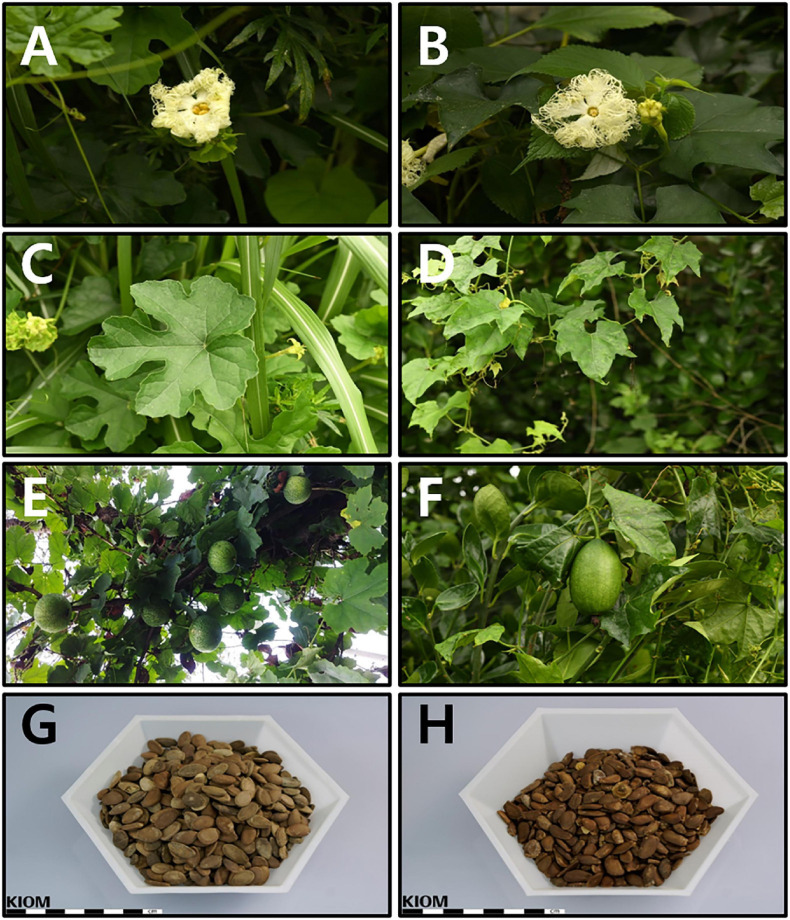
Characteristics of *Trichosanthes* (*kirilowii* var.) *kirilowii* and *T.* (*kirilowii* var.) *japonica* in Korea **(A)** Inflorescence of *T. kirilowii.* **(B)** Inflorescence of *T. japonica.* **(C)** Leaf shape of *T. kirilowii.* **(D)** Leaf shape of *T. japonica.* **(E)** Fruit of *T. kirilowii.* **(F)** Fruit of *T. japonica.* **(G)** Seeds of *T. kirilowii.* **(H)** Seeds of *T. japonica.*

The floral parts (female and male flowers) were remarkably similar (Figures 1A,B). However, leaf lobation patterns were clearly distinct. The leaves of *T. kirilowii* var. *kirilowii* were deeply (about half to two-thirds) 5-lobed (rarely 3- or 7-lobed) with a rhombic-obovate to oblong-shaped middle lobe (Figure 1C). In contrast, the leaves of *T. kirilowii* var. *japonica* were shallowly (up to the middle) 3-lobed, rarely almost unlobed or 5-lobed, with an unlobed and triangular to triangular-ovate middle lobe (Figure 1D). In the case of the fruit, pepos were globose to oblong in *T. kirilowii* var. *kirilowii* (Figure 1E), and oblong to ellipsoidal in *T. kirilowii* var. *japonica* (Figure 1F). Both had oblong- to oblong-ovate-shaped seeds, but seed color was somewhat different. Based on the RHS Color Chart, *T. kirilowii* var. *kirilowii* seeds were bright orange/brown (RHS Color Chart 164A, B to 165A, B; Figure 1G), whereas *T. kirilowii* var. *japonica* seeds were a dark orange/brown (RHS Color Chart 174A, B to 175A, B; Figure 1H).

### *Trichosanthes* Plastid Genome Organization

**FIGURE 2:**
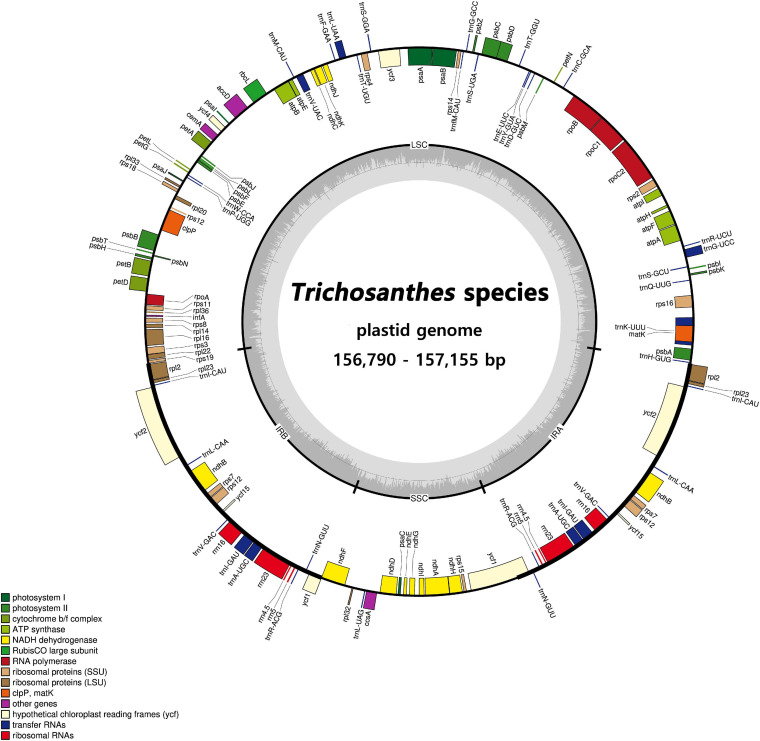
Circular gene map of plastid genomes from *Trichosanthes* species. Genes drawn inside the circle are transcribed clockwise, and those outside the circle are transcribed counterclockwise. The darker gray in the inner circle represents GC content. The gene map corresponds to the *T. kirilowii* chloroplast genome.

Sequencing generated 1.5–1.8 Gb raw paired-end read data and 1.1–1.5 Gb trimmed reads (Supplementary Tables 8, 9). We obtained two to four initial plastid contigs from all the assembled contigs (Supplementary Figure 2). The complete plastid genomes of the *Trichosanthes* accessions had mean read depths of 570×, 547×, 852×, 303×, and 84×. They varied in length from 156,790 to 157,155 bp, and all had a typical quadripartite structure with an LSC region of 86,047–86,242 bp, SSC region of 18,219–18,341 bp, and IR regions of 86,048–86,243 bp (Figure 2, Table 1 and Supplementary Figure 3). The GC content was 37.1% in all five accessions. In general, the GC content of the IR (42.8%) was higher than those of the LSC (34.8–34.9%) and the SSC (31.0–31.2%) (Table 1). All *Trichosanthes* plastid genomes had 113 genes, including 79 protein-coding, four rRNA, and 30 tRNA genes (Table 2). They had 18 intron-containing genes, with three duplicate genes (*ndhB*, *trnI-GAU*, and *trnA-UGC*) in the IR regions, 16 of which had a single intron and two of which (*ycf3* and *clpP*) had three introns (Supplementary Table 10). The *infA* gene was probably a pseudogene, with an early stop codon in all five plastid genomes. To identify IR contractions and extensions, we analyzed the IR boundaries of five *Trichosanthes* accessions in comparison to *H. macrocarpa* (Supplementary Figure 4). *Rps19* was confined to the LSC in *H. macrocarpa* but extended into the IR regions in *Trichosanthes*. The *ycf1* and *ndhF* genes were located at the IRa/SSC junction. The *rpl2* genes of all species were located in the IRa region, with part of the gene duplicated in IRb. Analysis of codon usage and anticodon recognition patterns indicated that the five *Trichosanthes* accession plastid genomes contained 26,737–26,769 codons, and leucine, isoleucine, and serine were the most abundant (Supplementary Figure 5). The relative synonymous codon usage (RSCU) values indicated synonymous codon usage bias with high proportions of A or T in the third position. To identify codon patterns in Cucurbitaceae plastid genomes, we analyzed codon distribution in 33 plastid genomes using the hierarchical clustering method (Supplementary Figure 6). RSCU values below 1.00 indicated cases where codons were used less frequently than expected, whereas RSCUs values above 1.00 indicated cases were codons were used more frequently than expected. In Supplementary Figure 6, the colors green and red indicate strong (RSCU value > 1) and weak (RSCU value < 1) codon usage bias, respectively. The codons with an A or T in the third position had a strong codon bias. Most RSCU values had similar patterns in Cucurbitaceae. AGA (arginine) usually had high RSCU values. However, *Gynostemma* had genus-specific patterns–e.g., AGG (arginine) yielded particularly low RSCU values.

**TABLE 1:** Features of *Trichosanthes* plastid genomes.

Species	*T. kirilowii* var. *kirilowii* 1	*T. kirilowii* var. *kirilowii* 2	*T. kirilowii* var. *japonica* 1	*T. kirilowii* var. *japonica* 2	*T. rosthornii*
Accession number	MT211646	MT211647	MT211648	MT211649	MT211650
Total plastid genome size (bp)	156,797	156,790	157,155	157,066	157,097
Large single copy (LSC) region (bp)	86,047	86,047	86,240	86,187	86,242
Inverted repeat (IR) region (bp)	26,262	26,262	26,303	26,285	26,257
Small single copy (SSC) region (bp)	18,226	18,219	18,309	18,309	18,341
Total number of genes (unique)	113	113	113	113	113
Protein-coding (unique)	79	79	79	79	79
rRNA (unique)	4	4	4	4	4
tRNA (unique)	30	30	30	30	30
GC content (%)	37.1	37.1	37.1	37.1	37.1
LSC (%)	34.9	34.9	34.8	34.8	34.9
IR (%)	42.8	42.8	42.8	42.8	42.8
SSC (%)	31.2	31.2	31.0	31.0	31.1

**TABLE 2:** Genes in the plastid genomes of *Trichosanthes* species.

Gene groups	Gene names
Photosystem I	*psaA, B, C, I, J, ycf3^2)^, ycf4*
Photosystem II	*psbA, B, C, D, E, F, H, I, J, K, L, M, N, T, Z*
Cytochrome b6/f	*petA, B^1)^, D^1)^, G, L, N*
ATP synthase	*atpA, B, E, F^1)^, H, I*
Rubisco	*rbcL*
NADH oxidoreductase	*ndhA^1)^, B^1)^^3)^, C, D, E, F, G, H^3)^, I, J, K*
Large subunit ribosomal proteins	*rpl2^1)3)^, 14, 16^1)^, 20, 22, 23^3)^, 32, 33, 36*
Small subunit ribosomal proteins	*rps2, 3, 4, 7^3)^, 8, 11, 12 ^2)^^3)^^4)^, 14, 15^3)^, 16^1)^, 18, 19*
RNA polymerase	*rpoA, B, C1^1)^, C2*
Unknown function protein-coding gene	*ycf1^3)^, 2^3^, 15^3)^*
Other genes	*accD, ccsA, cemA, clpP^2)^, matK*
Ribosomal RNAs	*rrn16^3)^, 23^3)^, 4.5^3)^, 5^3)^*
Transfer RNAs	*trnA–UGC^1)3)^, trnC–GCA, trnD–GUC, trnE–UUC, trnF–GAA, trnfM–CAU, trnG–GCC, trnG–UCC, trnH–GUG, trnI–CAU^3)^, trnI–GAU^1)3)^, trnK–UUU, trnL–CAA^3)^, trnL–UAA, trnL–UAG, trnM–CAU, trnN–GUU^3)^, trnP–UGG, trnQ–UUG, trnR–ACG^3)^, trnR–UCU, trnS–GCU, trnS–GGA, trnS–UGA, trnT–GGU, trnT–UGU, trnV–GAC^3)^, trnV–UAC, trnW–CCA*, and *trnY–GUA*

*1) Gene contains a single intron, 2) gene contains two introns, 3) two gene copies in IRs, 4) trans-splicing gene.*

### SSR and Tandem Repeat Analysis in *Trichosanthes* Plastid Genomes

**FIGURE 3:**
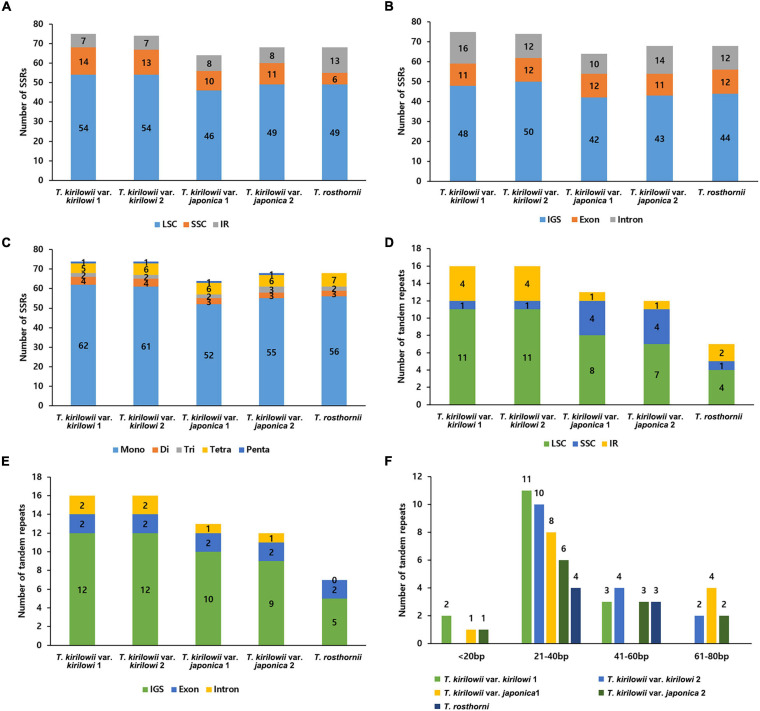
Distribution of repeat sequences in the five *Trichosanthes* plastid genomes. **(A)** Number of SSRs in genomic regions. **(B)** Distribution of SSRs in intergenic spacer (IGS), exon, and intron regions. **(C)** Distribution of SSR types. **(D)** Distribution of tandem repeats in genomic regions. **(E)** Distribution of tandem repeats in intergenic spacer (IGS), exon, and intron regions. **(F)** Distribution of tandem repeat lengths.

The five *Trichosanthes* accessions had 64–75 SSRs in total (Figure 3A). Most SSRs were in the LSC and SSC regions within intergenic spacers (Figures 3A,B). The mononucleotide motif was the most abundant in all the accessions (Figure 3C). No hexanucleotide motif repeats were found. Most tandem repeats were located in the LSC (Figure 3D). *T. kirilowii* var. *kirilowii* and *T. kirilowii var. japonica* had tandem repeats in different genomic regions (Figure 3E). *T. rosthornii* had fewer tandem repeats than other *Trichosanthes* taxa. There were 7–16 tandem repeats of >20 bp identified in the five *Trichosanthes* accessions, and most were 21–40 bp long (Figure 3F).

### Comparative Analysis of *Trichosanthes* Plastid Genomes

**FIGURE 4:**
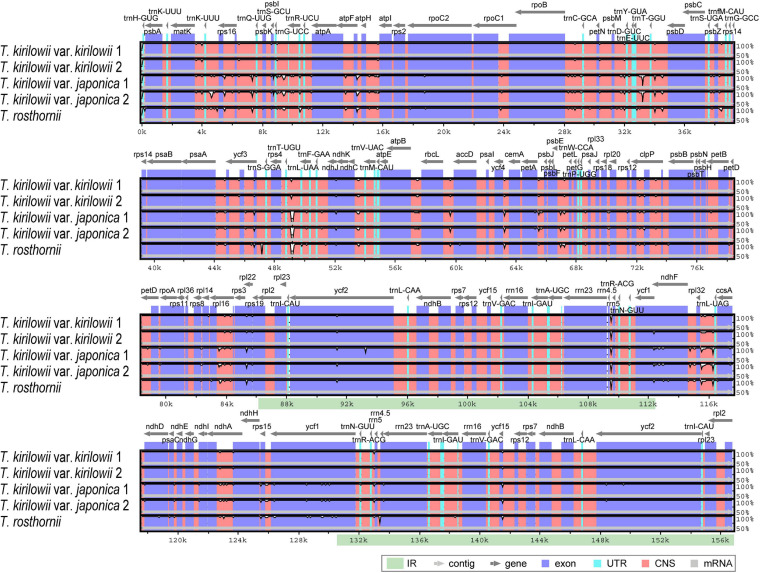
Comparison of *Trichosanthes* plastid genomes using mVISTA. Complete plastid genomes of five *Trichosanthes* species were compared, with *T. kirilowii* var. *kirilowii* 1 as the reference. The gray arrows above the alignment indicate genes. Different colors represent different regions (coding and non-coding). The horizontal axis indicates the coordinates within the chloroplast genome. The vertical scale represents the percentage of identity, ranging from 50 to 100%.

**FIGURE 5:**
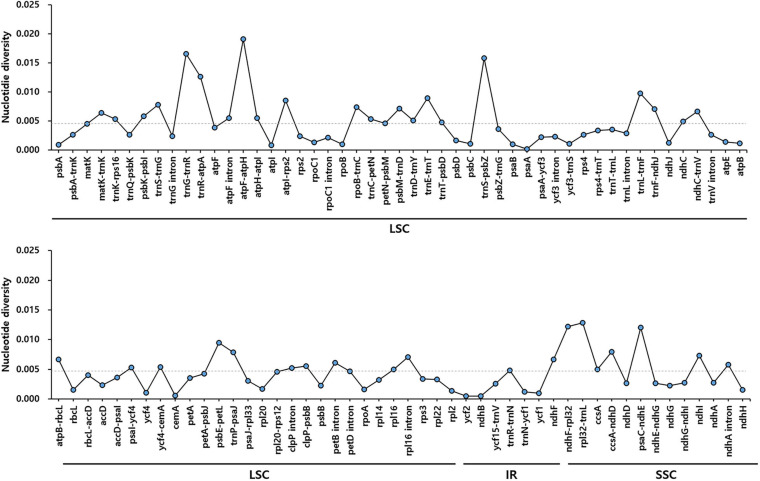
Comparison of nucleotide diversity (Pi) values among the five *Trichosanthes* species. The dotted line indicates the average Pi value by gene and intergenic region in five *Trichosanthes* plastid genomes. Regions with Pi = 0 were excluded.

To identify divergent regions, we analyzed the plastid genomes of all five *Trichosanthes* accessions using the mVISTA program with *T. kirilowii* var. *kirilowii* as a reference (Figure 4). Overall, the alignment revealed that IR regions were better conserved than the single-copy regions, and intergenic regions were more divergent than genic regions, except for *ycf1*, *ycf2*, *rps3*, and *rpl22*. The major regions of divergence (including less than 50% similarity) were identified in the intergenic regions (IGS) *trnT-UGU–trnL-UAA*, *trnE-UUC–trnT-GGU, rrn4.5–rrn5*. The differences observed between *T. kirilowii* var. *kirilowii* and *T. kirilowii* var. *japonica* in the regions could indicate that they are two different species. To determine sequence divergence in the five *Trichosanthes* accessions, we calculated the nucleotide diversity as a Pi value (Figure 5). The IGS region in *atpF-atpH* exhibited high divergence, with a Pi of 0.0198. In the genic region, the value of Pi for *ndhC* was 0.00496, indicating that the genic region was more highly conserved than the IGS region, as expected. In the present study, although the plastid genomes of *Trichosanthes* exhibited a highly conserved structure, highly local sequence variability was detected in IGS.

### New Molecular Markers for Distinguishing *Trichosanthes* Species

We compared the nuclear ITS sequences of *T. kirilowii* var. *kirilowii*, *T. kirilowii* var. *japonica*, *T. rosthornii*, and *T. rubriflos*. We did not find any variation among *T. kirilowii* and *T. rosthornii* (Supplementary Figure 7). Only *T. kirilowii* var. *japonica* and *T. rubriflos* had a few differences compared to the other species, showing the limitation of this universal DNA barcode in distinguishing *Trichosanthes* at the species level and below. To address the problem, we developed a marker set to distinguish *T. kirilowii* var. *kirilowii*, *T. kirilowii* var. *japonica*, and *T. rosthornii* using indels. Three intergenic regions (*trnT-UGU–trnL-UAA*, *rrn4.5–rrn5*, and *trnE-UUG–trnT-GGU*) were revealed to be considerably helpful by containing species-specific indels (Supplementary Figure 8).

**FIGURE 6:**
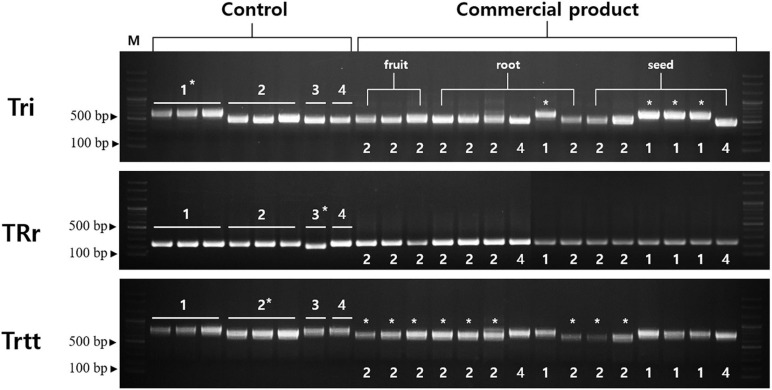
Gel images of indel markers for *Trichosanthes*. Primers for Tri, TRr, and Trtt were tested with 15 commercial products on the Korean market. Control samples were used in a marker test (Supplementary Table 4). Information regarding the commercial products is provided in Supplementary Table 5. An asterisk indicates matching between band patterns of control samples and commercial products. 1, *Trichosanthes kirilowii* var. *kirilowii*; 2, *Trichosanthes kirilowii* var. *japonica*; 3, *Trichosanthes rosthornii*; 4, *Trichosanthes rubriflos*.

Tri, TRr, and Trtt markers were successfully amplified for all samples (Figure 6). The Tri marker (located in the *trnT-UGU–trnL-UAA* region) differentiated *T. kirilowii* var. *kirilowii* from *T. kirilowii* var. *japonica, T. rosthornii*, and *T. rubriflos*. The Tri marker was 640, 512, and 502 bp long in *T. kirilowii* var. *kirilowii*, *T. kirilowii* var. *japonica*, and *T. rosthornii*, respectively. The TRr marker (located in the *rrn4.5–rrn5* region) contained a 32-bp deletion in *T. rosthornii*, discriminating this species from the others. The Trtt marker (located in the *trnE-UUG–trnT-GGU* region) contained an 88-bp deletion in *T. kirilowii* var. *japonica* compared to the other *Trichosanthes* (Figure 6 and Supplementary Figure 8). Therefore, the four *Trichosanthes* taxa could be unambiguously discriminated using the three markers developed in the present study (Figure 6). Furthermore, we surveyed 15 commercial drugs made of crude *Trichosanthes* tissues from South Korea (Supplementary Table 5). Only four products were identified as *T. kirilowii* var. *kirilowii* using the Tri marker; no products were identified as *T. rosthornii* using the TRr marker. Nine products were identified as *T. kirilowii* var. *japonica* using Trtt. The two products without marked asterisks on Figure 6 were inferred to be *T. rubriflos* through the simultaneous use of all three markers (Tri, TRr, and Trtt). All fruit products were identified to be *T. kirilowii* var. *japonica*, while root and seed products were identified to be *T. kirilowii* var. *japonica* (four root and two seed products), *T. rubriflos* (one root and one seed product) or *T. kirilowii* var. *kirilowii* (one root and three seed products).

### Phylogenetic Relationships Among *Trichosanthes* Species Within Cucurbitaceae

**FIGURE 7:**
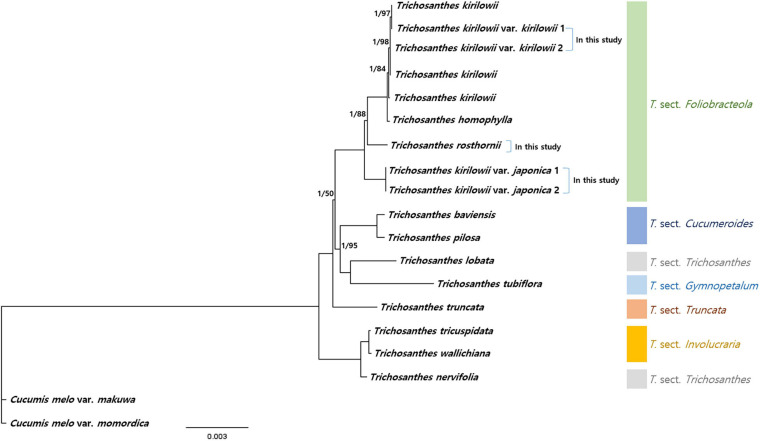
Phylogenetic relationships of *Trichosanthes*. Bayesian Inference (BI) topology is shown. The numbers above branches represent BI posterior probability, and Maximum Likelihood (ML) bootstrap support; branches without support values have maximum support (PP 1, BS 100).

To verify the phylogenetic relationships among *Trichosanthes*, we identified 73 protein-coding sequences (60,681 bp in total) shared by our five *Trichosanthes* accessions and 14 other *Trichosanthes* accessions, with *Cucumis melo* subsp. *melo* and *Cucumis melo* var. *momordica* as the outgroup (Figure 7 and Supplementary Figure 9). Phylogenetic relationships inferred from BI and ML were essentially identical (Figure 7 and Supplementary Figure 9). All phylogenetic relationships were strongly supported (BI PP = 1.0). *Trichosanthes* formed a monophyletic group with the exception of, *T. nervifolia* that is grouped with *T.* sect. *Involucraria* instead of with the other included species of sect. *Trichosanthes*. *T. kirilowii* var. *kirilowii* and *T. kirilowii* var. *japonica* were clustered with *T. rosthornii* and *T. homophylla* into *T.* sect. *Foliobracteola*. Whereas *T. kirilowii* var. *kirilowii* and *T. kirilowii* var. *japonica* were monophyletic, *T. kirilowii* as a whole was paraphyletic due to the nested positions of *T. rosthornii* and *T. homophylla*.

## Discussion

### Features of *Trichosanthes* Plastid Genomes

In the present study, we newly determined the complete plastid genomes of five *Trichosanthes* accessions. Of those, *T. kirilowii* var. *japonica* and *T. rosthornii* were sequenced for the first time. Plastid genomes in *Trichosanthes* have 113 unique genes and their gene order, GC contents, genomic structure, and overall length (156,790–157,155 bp) are within the ranges described previously for angiosperm plastid genomes (Millen et al., 2001). *Trichosanthes* plastid genomes have one pseudogene, *infA*, which is the result of an early stop codon. The gene *infA* frequently contains deletions and early stop codons in angiosperm plastid genomes (Wicke et al., 2011). It has been transferred from plastid genomes to the nucleus in many plants. The *infA* encoded translation initiation factor was independently lost during land plant evolution (Wicke et al., 2011; Menezes et al., 2018). Codon usage is an essential factor for the expression of genetic information correctly, and it plays an important role in shaping plastid genome evolution (Wang et al., 2016; Yi et al., 2018). High relative synonymous codon usage (RSCU) values correspond to more highly conserved plastid genes (Wang et al., 2016; Ivanova et al., 2017; Zuo et al., 2017). The five *Trichosanthes* accessions have nearly the same codons, which are similar to those of other plastid genomes (Wang et al., 2016; Raman et al., 2019). We also surveyed the RSCU values of 28 other Cucurbitaceae plastid genomes. Half of the codons had a high codon bias (Supplementary Figure 6) and are denoted in green in the figure (RSCU > 1). This result is similar to those of other plastid genomes, and most codons with high RSCU values had an A or T in the third position of their amino acid. The plastid genomes of *Gynostemma* species have codon bias patterns that are slightly different from those of other Cucurbitaceae. In particular, AGG (arginine) codons had relatively low RSCU values. The RSCU values of the *Trichosanthes* species and other Cucurbitaceae plastid genomes were similar to those of other higher plants.

### Repeat Sequences in *Trichosanthes* Plastid Genomes

Simple sequence repeats or microsatellites of 1–6 nucleotides are widely distributed throughout genomes (Curci et al., 2015). They are useful in population genetics studies and for discriminating species, and facilitate phylogenetic studies due to their high polymorphism at the intra- and interspecific levels (Powell et al., 1995; Zalapa et al., 2012). In the present study, mononucleotide SSRs comprised approximately 83% of all SSRs (55–62 SSRs), which were mostly detected in IGS regions (Figure 3). The finding is similar to those of previous reports, in which most of the mononucleotide repeats were A and T repeats due to an abundance of polyamines and polythymines in the plastid genome (Qian et al., 2013; Yang et al., 2016; Wang et al., 2018). SSRs identified in the *Trichosanthes* plastid genome could provide useful genetic resources for *Trichosanthes* species identification and population genetics studies. Repeat sequences facilitate phylogenetic research and species identification (Asaf et al., 2016). Tandem repeats, 7–16 nucleotides long, were detected in the five *Trichosanthes* plastid genomes. Among the five *Trichosanthes* taxa, *T. rosthornii* had the fewest tandem repeat sequences. *T. kirilowii* var. *kirilowii* and *T. kirilowii* var. *japonica* exhibited differences in SSRs and tandem repeats. In general, repeats are very similar between individuals of a similar species (Park et al., 2018b), but the two *T. kirilowii* varieties are completely different in terms of repeats. Furthermore, we did not observe a clear difference in the tandem repeat copy number, as was seen in previous studies for distinguishing species (Hong et al., 2017; Park et al., 2018b); however, a general repeat sequence variation was observed (Park et al., 2017). Such repeat sequences serve as molecular markers in population genetics and phylogenetic studies of Cucurbitaceae, including *Trichosanthes*.

### Genetic Variation in the *Trichosanthes* Accessions

According to the mVISTA results, the plastid genome of *Trichosanthes* has low diversity, and its genic regions are more conserved than its IGS regions, the latter of which are consistent with angiosperm plastid genomes in general (Shaw et al., 2007; Huo et al., 2019; Song et al., 2019). The *trnT*–*trnL*, *rrn4.5–rrn5*, and *trnE–trnT* regions were observed to be hotspot regions for genetic variation (Figure 4). The hotspot regions in plant species are caused by mutation events (Morton and Clegg, 1993; Maier et al., 1995; Liu et al., 2018, 2019), and can be used as DNA barcodes to distinguish species or genera, depending on the variability of the regions. Such regions have been successfully used for the development of molecular markers to efficiently distinguish species (Cho et al., 2015; Hong et al., 2017; Park et al., 2018a, b). In terms of nucleotide diversity (Pi), most divergent regions were non-coding, and this is consistent with other plastid genomes, which have been reported to have highly variable non-coding regions at *trnG–trnR*, *trnR–atpA*, *atpF–atpH*, *trnS–psbZ*, *trnL–trnF*, *ndhC–trnV*, *psbE–petL*, *ndhF–rpl32*, and *rpl32–trnL* (Wang et al., 2010; Schroeder et al., 2016; Zhitao et al., 2017; Liu et al., 2019; Smidt et al., 2020).

Inverted repeat (IR) contraction and expansion in angiosperm plastid genomes cause plastid genome size to vary (Raubeson et al., 2007). Previous studies have identified extremely short IRs, or the loss of IR regions and genes (Wang et al., 2008; Zhu et al., 2016). Compared to *H. macrocarpa*, *Trichosanthes* exhibits a highly conserved IR length and gene positions. However, The IR region length ranged from 26,257 to 26,303, meaning that there was some contraction/expansion in the IR region (Supplementary Figure 4). This phenomenon has also been observed in other Cucurbitaceae plastid genomes (Zhang et al., 2018; Bellot et al., 2020).

### Molecular Marker Development and Commercial Products Screening in Korean Herbal Markets

Authentication of herbal materials is essential for quality control, safety, and herbal medicine efficacy. Medicinal plants are extensively used for disease prevention, side effects management, and for their pharmacological effects (Yu et al., 2018). Adulterants in herbal medicine could have similar morphology, uncertain origins, and often bear names that are similar to those of the original ingredient (Han et al., 2016; Ichim, 2019). Their use may bring about negative side effects and quality problems. In Korean herbal medicine, the roots and seeds of *T. kirilowii* and *T. rosthornii* are considered authentic Trichosanthis Radix and Trichosanthis Semen, respectively (Korea Institute of Oriental Medicine [KIOM], 2020). However, *T. kirilowii* var. *kirilowii* and *T. kirilowii* var. *japonica*, distributed in South Korea, appear highly morphologically similar to the naked eye, and therefore, are often misused or used interchangeably.

In the present study, we developed an indel marker set to facilitate the distinction of authentic and adulterated *Trichosanthes* materials. Three novel markers–Tri, TRr, and Trtt–can completely discriminate *T. kirilowii* var. *kirilowii* from *T. kirilowii* var. *japonica*, *T. rosthornii*, and *T. rubriflos* (Figure 6). We also tested commercial products in South Korea. Among the 15 product samples tested, only four were composed of *T. kirilowii*, and no *T. rosthornii* was found, indicating that only the four were authentic. Most commercial products are prepared from *T. kirilowii* var. *japonica*, the adulterant. In the present study, we encountered problems in the context of quality control for authentic herbal medicines. The novel indel markers developed in the present study will facilitate rapid and accurate authentication of *Trichosanthes* herbal medicines, as well as the determination of whether they have been adulterated. Consequently, further studies are required to test the quality of the numerous commercial products available in markets.

### Phylogenetic Relationships Among *Trichosanthes* and Taxonomic Identity of the *T. kirilowii* Complex

Our reconstructed phylogeny of the genus *Trichosanthes* is generally consistent with a recent infrageneric classification by de Boer and Thulin (2012). However, *T. nervifolia*, which currently is placed in sect. *Trichosanthes* together with *T. cucumerina* (de Boer et al., 2012), was instead closely related to taxa in sect. *Involucraria*. This topological conflict between plastome and nrDNA phylogenies may be due to chloroplast capture but additional morphological, micromorphological, anatomical, and palynological characteristics are needed to specify the exact sectional position of *T. nervifolia*. Considering the results of previous studies and those of the present study, the two infra-specific taxa of *T. kirilowii* have clear genetic differences that are large enough to distinguish them as separate species based on the phylogenetic species concept, as also concluded by de Boer et al. (2012) and Liu et al. (2021). Moreover, their habitats are different. *T. kirilowii* var. *kirilowii* is widely distributed across the temperate and subtropical regions of East Asia in inland habitats, such as forests, shrublands, and grasslands (Huang et al., 2011), whereas *T. kirilowii* var. *japonica* is rather narrowly distributed in Korea and Japan in islands and coastal regions (Ohba, 1999; Kim and Choi, 2018; Supplementary Figure 7). Finally, the two infra-specific taxa were clearly identified based on combined morphological characters such as leaf and fruit shape, and seed color according to the taxonomic concept of Regel (1868). We therefore propose that *T. kirilowii* var. *japonica* (≡*T. kirilowii* subsp. *japonica*) be recognized again as a separate species considering its distinct genetics, morphology, and geographical distribution (Huang et al., 2009; de Boer et al., 2012; Liu et al., 2021). Further molecular identification and in-depth phylogenetic studies, as well as morphological studies, using various and abundant samples of *Trichosanthes* are required to identify the unique traits among the species to facilitate their identification and classification.

From an herbal medicine perspective, *T. kirilowii* and *T. rosthornii* form a monophyletic clade, and *T. japonica* was separated from them. Therefore, the use of *T. japonica* as a medicinal herb is not recommended because its effects are unknown. The results of the present study offer data on the authenticity of Korean herbal medicine resources, particularly *Trichosanthes*, which could enhance the quality and safety of *Trichosanthes* herbal medicines.

## Conclusion

The present study identified the distinct morphological traits between *T. kirilowii* s. str (=*T. kirilowii* var. *kirilowii*) and *T. japonica* (=*T. kirilowii* var. *japonica*). The plastid genomes of the five *Trichosanthes* accessions studied were highly conserved with respect to gene content, gene orientation, GC content, and local variations. Most divergences were detected in non-coding regions (*trnT–UGU–trnL–UAA*, *rrn4.5–rrn5*, *trnE–UUC–trnT–GGU*). Such hotspot regions were used to create a novel marker set that successfully discriminated *T. kirilowii* from *T. japonica* and *T. rosthornii* in commercial herbal medicine products. Overall, our results distinguish *T. kirilowii* and *T. japonica* based on complete plastid genomes, novel marker sets, and phylogenetic relationships. Furthermore, our results could facilitate herbal medicine quality control by enabling the authentication of herbal medicines containing *T. kirilowii* and *T. rosthornii*.

## Data Availability Statement

The datasets presented in this study can be found in online repositories. The names of the repository/repositories and accession number(s) can be found in the article/Supplementary Material.

## Author Contributions

IP designed the experimental framework, drafted and revised the manuscript, performed experiments, and carried out genome analysis. J-HS, SY, SC, and BM collected and identified plant materials. IP and BM revised the manuscript. All authors contributed to the experiments and approved the final manuscript.

## Conflict of Interest

The authors declare that the research was conducted in the absence of any commercial or financial relationships that could be construed as a potential conflict of interest.

## Publisher’s Note

All claims expressed in this article are solely those of the authors and do not necessarily represent those of their affiliated organizations, or those of the publisher, the editors and the reviewers. Any product that may be evaluated in this article, or claim that may be made by its manufacturer, is not guaranteed or endorsed by the publisher.

## Footnotes

1
http://misaweb.ipk-gatersleben.de/

2
https://blast.ncbi.nlm.nih.gov/Blast.cgi

3
https://www.geneious.com
