# Supplementary material for: Plastid Phylogenomic Data Offers Novel Insights Into the Taxonomic Status of the Trichosanthes kirilowii Complex (Cucurbitaceae) in South Korea
Source: Front Plant Sci. 2021 Jul 27;12:559511. doi: 10.3389/fpls.2021.559511 (PMC8353159; doi:10.3389/fpls.2021.559511)
Supplement: Supplementary file 1 [file Data_Sheet_1.docx]

Supplementary Material

Plastid phylogenomic data offers novel insights into the taxonomic status of the *Trichosanthes kirilowii* complex (Cucurbitaceae) in South Korea

Inkyu Park, Jun-Ho Song, Sungyu Yang, Sungwook Chae, Byeong Cheol Moon*

***Correspondence:** Byeong-Cheol Moon: bcmoon@kiom.re.kr

# Supplementary Figures and Tables


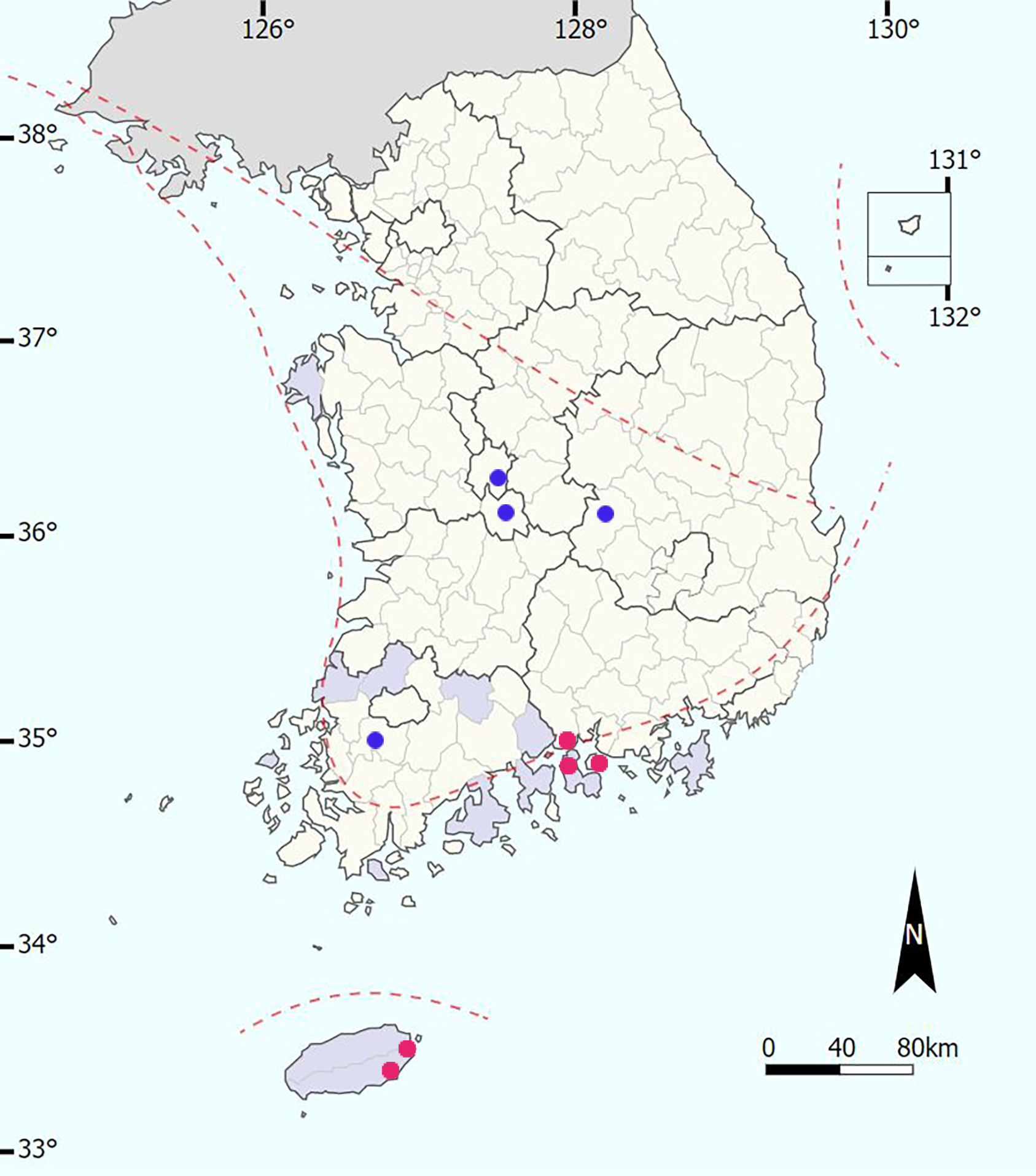


**Figure S1.** Distribution of studied samples of *Trichosanthes kirilowii* var. *kirilowii* (blue dots) and of *T. kirilowii* var. *japonica* (red dots); the shaded area shows the reported distribution of *T. kirilowii* var. *japonica*.

#
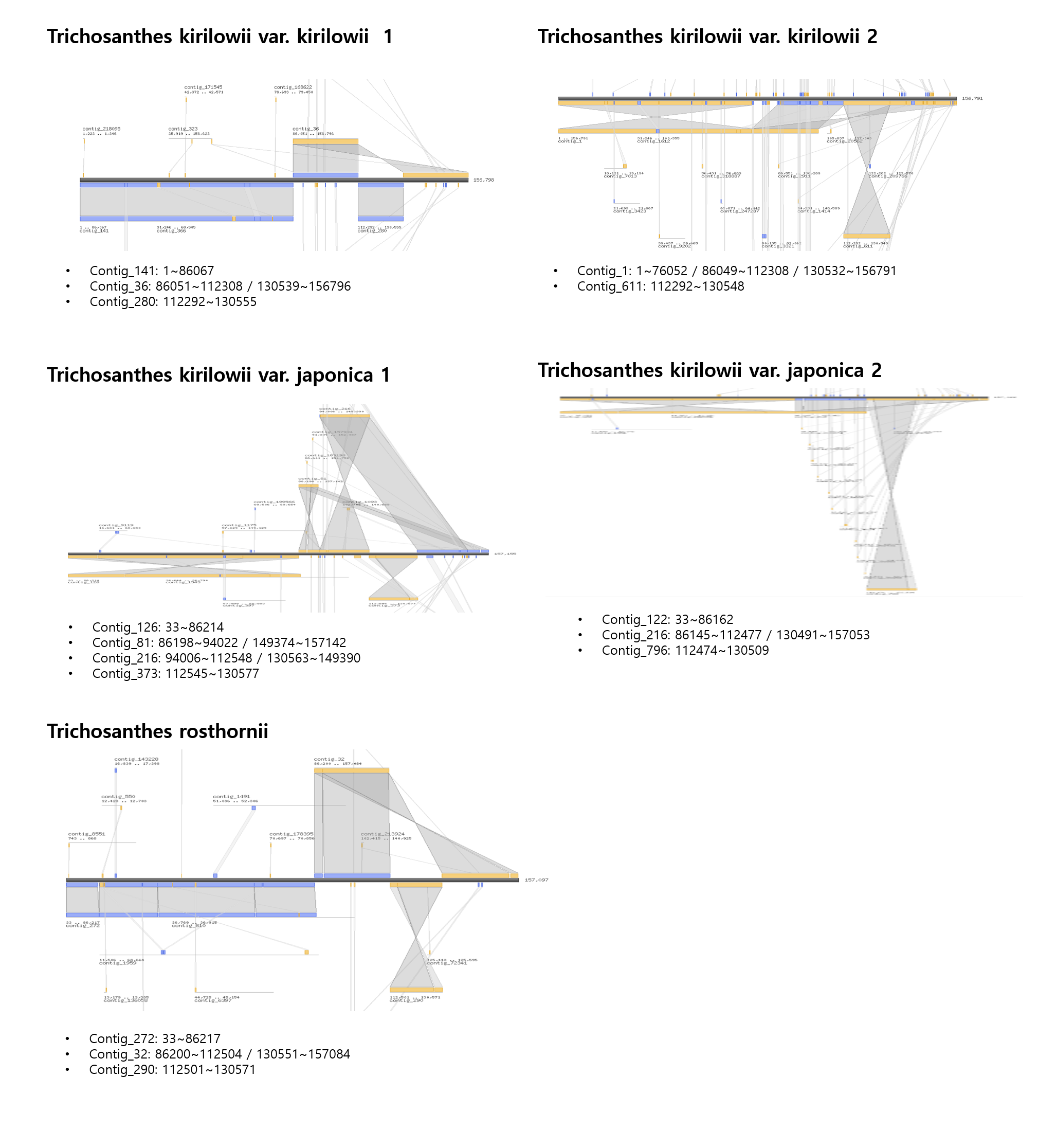


Figure S2 Alignment of the initial contigs representing the plastid genome on the complete plastid genome sequence. The contig numbers are indicated under the contigs and hit positions are under the reference plastid genome sequences for *Hodgsonia macrocarpa* (NC_039628).

#
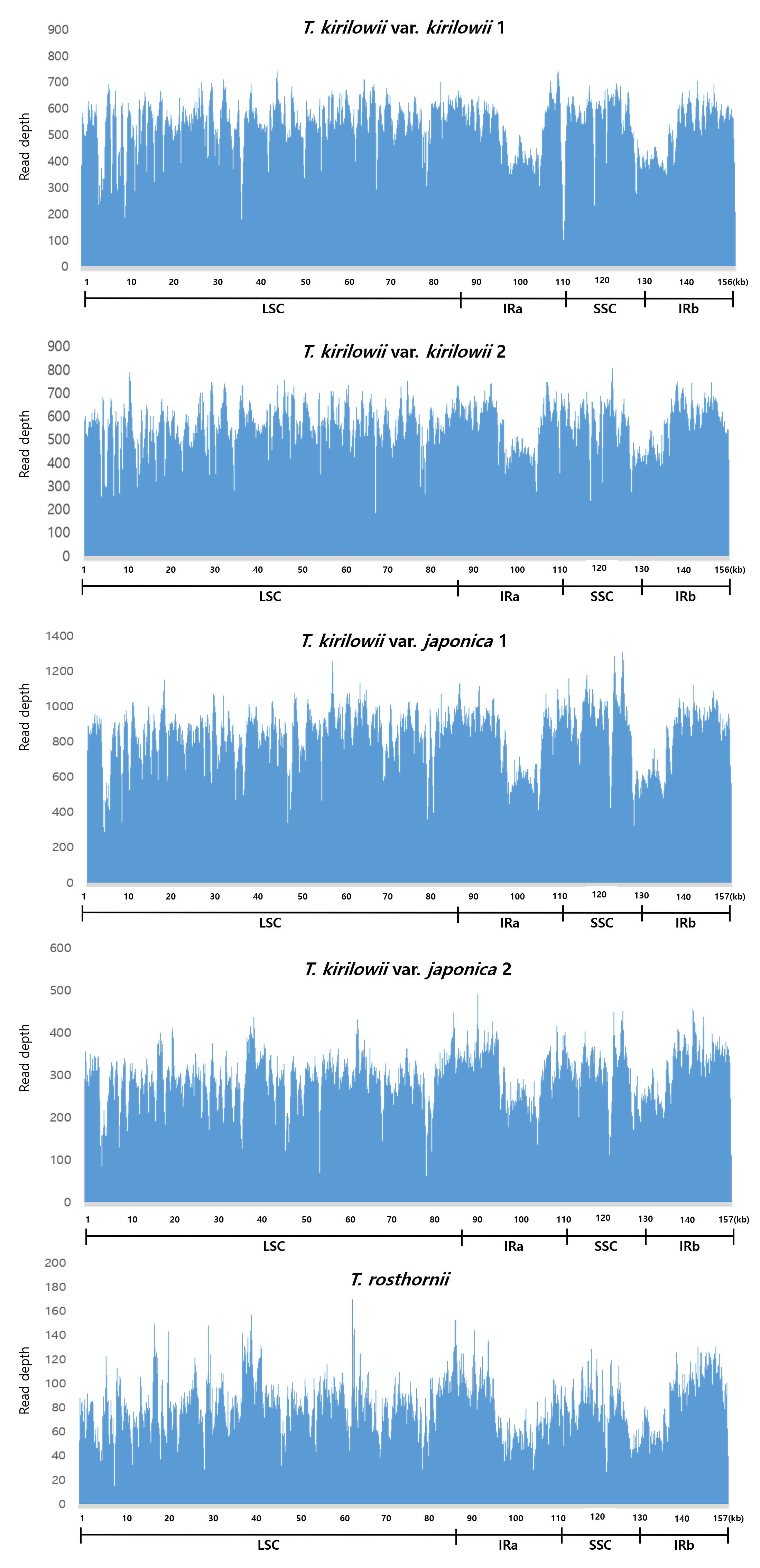


Figure S3 Distribution of paired-end reads mapped onto the complete plastid genomes of two accessions each of *Trichosanthes kirilowii* var. *kirilowii* and *T. kirilowii* var. *japonica* and one accession of *T. rosthornii*. LSC, large single-copy region; SSC, small single-copy region; IRa, inverted repeat a; IRb, inverted repeat b

Figure S4 Comparison of the LSC, IR, and SSC junction positions in *Trichosanthes* species and *Hodgsonia macrocarpa* plastid genomes. The bp indicate the distance or the overlap between IR boundaries and the closest gene.


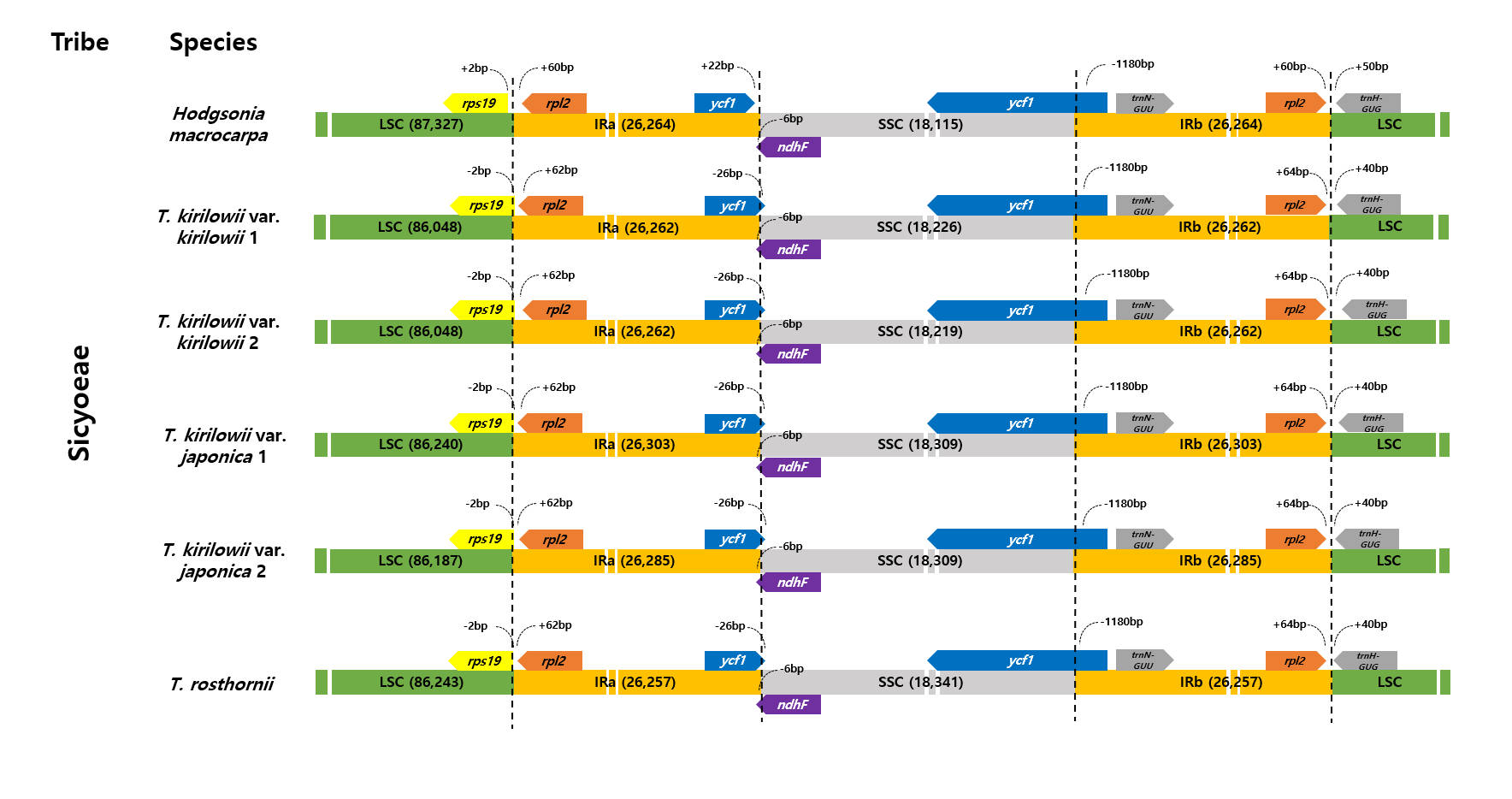


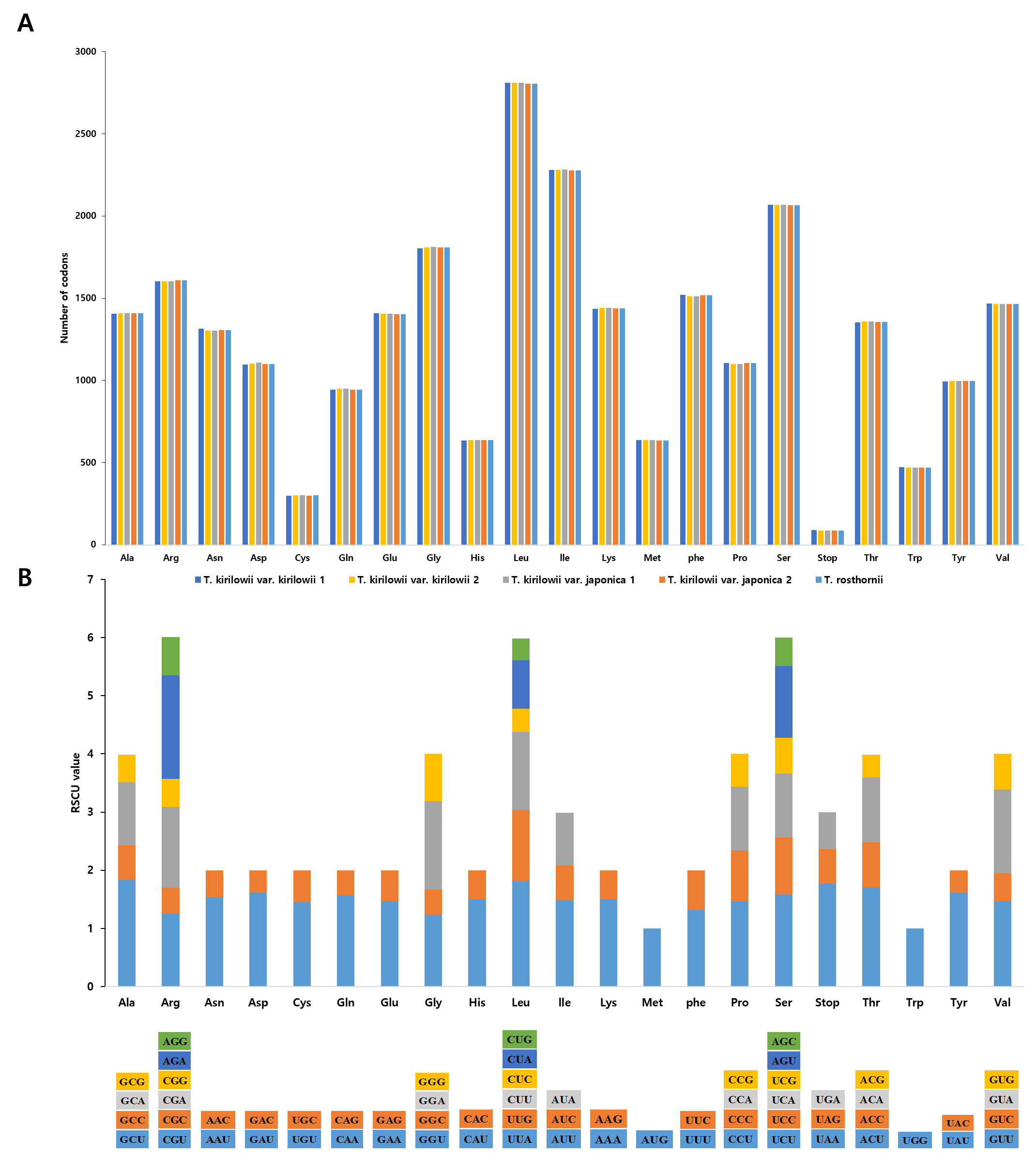


Figure S5 Codon frequencies and relative synonymous codon usage (RSCU) values for *Trichosanthes* plastid genomes. (A) Amino acid frequencies for 78 protein-coding sequences. (B) RSCU for 20 amino acids and stop codons in 78 protein-coding sequences.


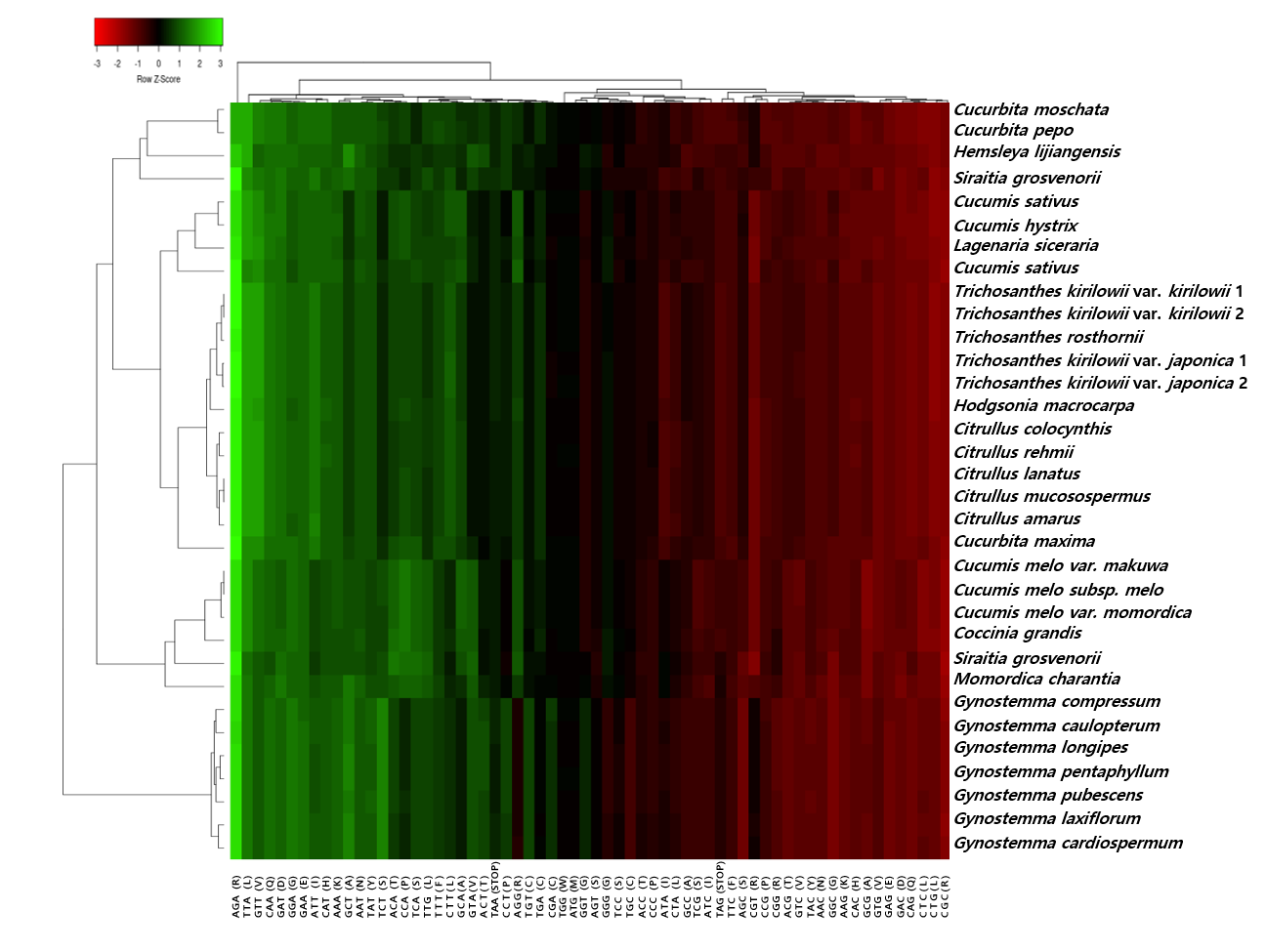


Figure S6 Codon distribution of protein-coding genes in Cucurbitaceae plastid genomes. Green indicates a high relative synonymous codon usage (RSCU) value and red indicates a low RSCU value. Hierarchical clustering (average linkage method) was performed for the codon patterns (x-axis). All RSCU values are normalized using the Z- score.


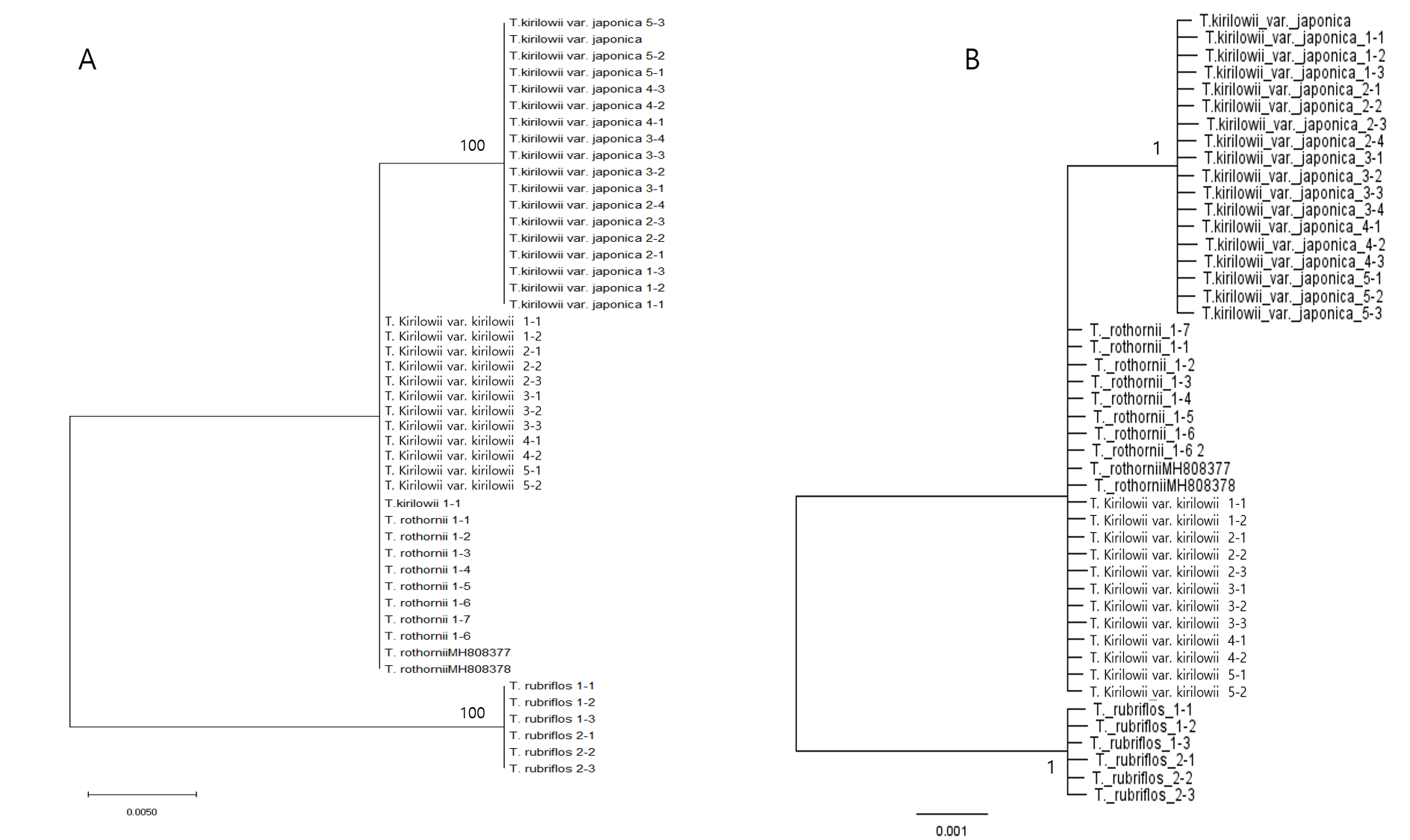


**Figure S7. Phylogenetic tree based on ITS from three *Trichosanthes* species using Maximum likelihood (A) and Bayesian Inference (B).** The same samples are shown in supplementary Table S1; additional specimens have been registered in the Korean Herbarium of Standard Herbal Resources (Index Herbarium Code KIOM) at the Korea Institute of Oriental Medicine (KIOM). MH808377 and MH808378 were downloaded from GenBank.**
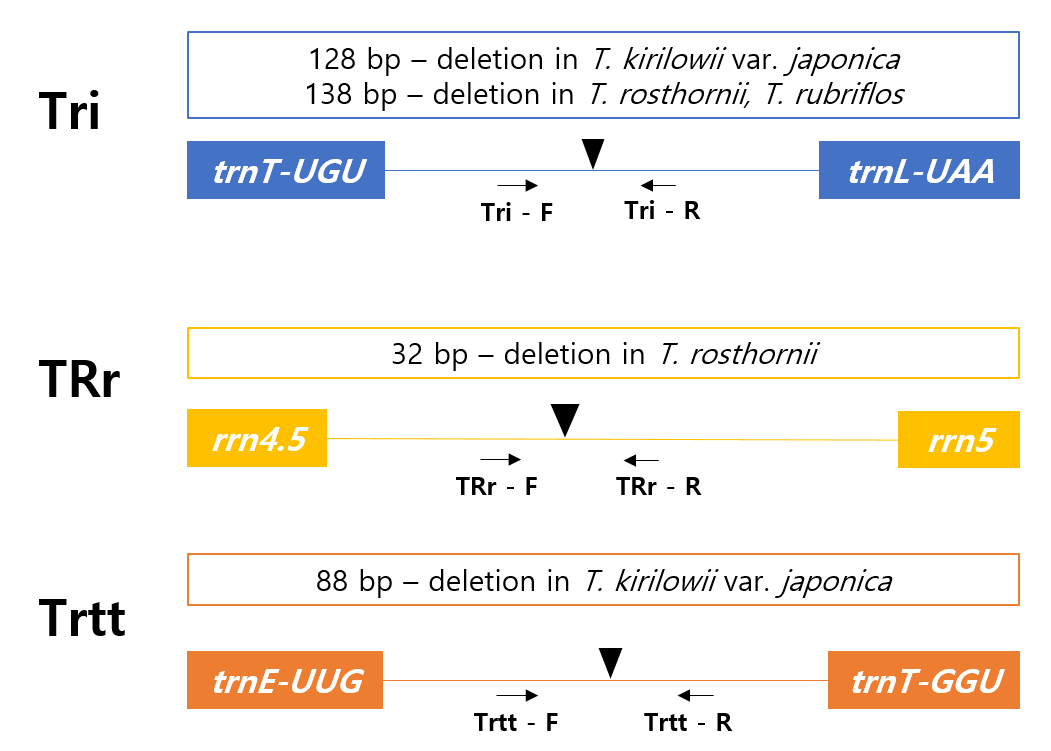
** **Figure S8. Schematic for diagnostic markers of three *Trichosanthes* taxa, *T. kirilowii* var. *kirilowii*, *T. kirilowii* var. *japonica* and *T. rosthornii*, showing their specific primer position and the indel variation.**


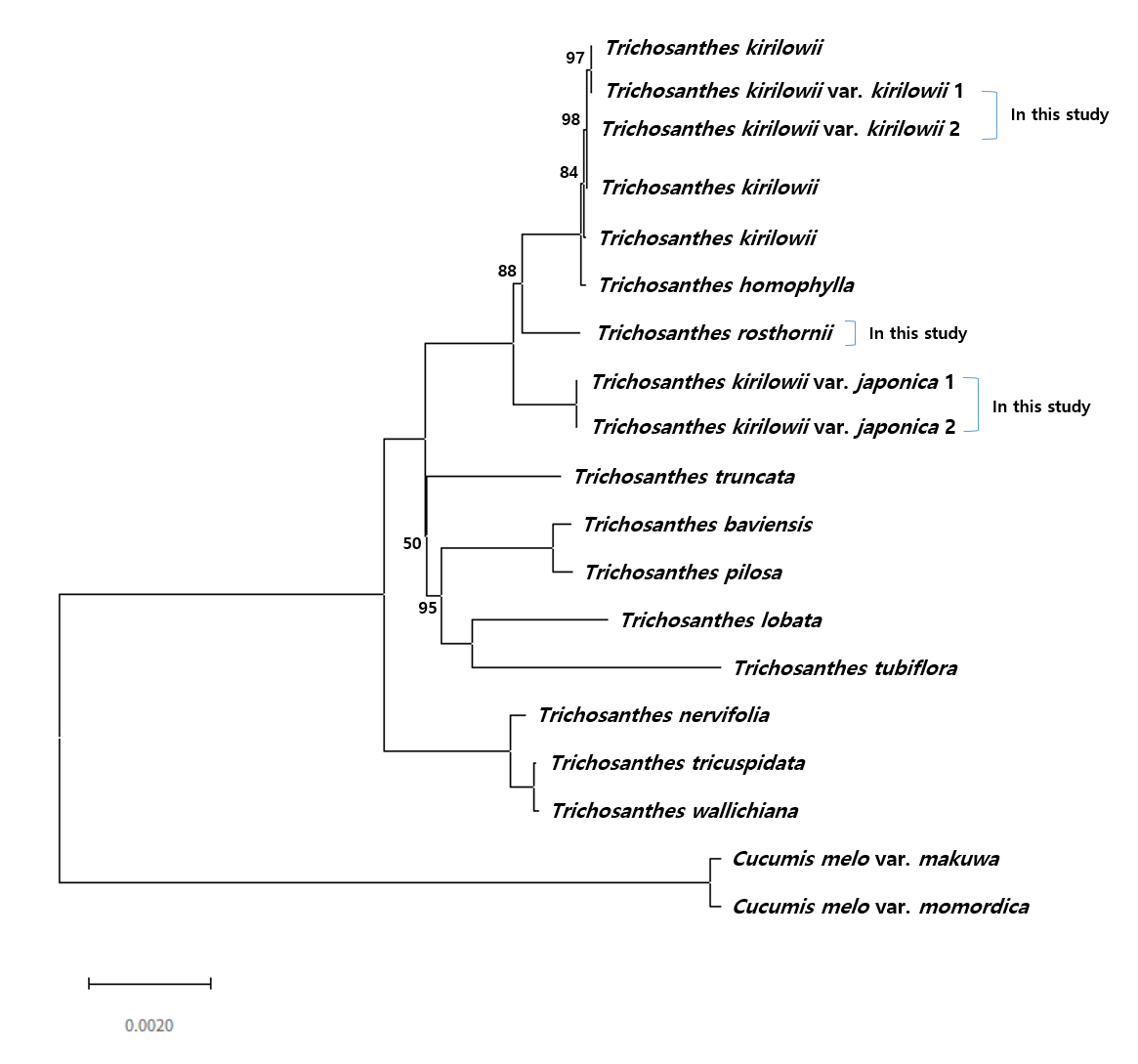


**Figure S9.** **Phylogenetic relationships of *Trichosanthes* inferred using maximum likelihood of 73 protein-coding genes; numbers at nodes are maximum likelihood bootstrap values (BS); branches without support values have maximum support (BS 100).**

**Table S1 Voucher specimen information for morphology and chloroplast genomes used in this study**

| No. | Species | Collection information | **Coordinates** | Collector *collection no.* (Herbarium acronym) |
| --- | --- | --- | --- | --- |
| 1 | *Trichosanthes kirilowii* var. *kirilowii* | Cheongju-si,  Chungcheongbuk-do, Korea | 36°32'32.3"N 127°28'50.3"E | B.C. Moon *2017-69-1* (KIOM)^C, M, B^ |
|  |  |  |  | B.C. Moon *2017-69-2* (KIOM)^M^ |
|  |  |  |  | B.C. Moon *2017-69-3* (KIOM)^M^ |
|  |  |  |  | B.C. Moon *2017-69-4* (KIOM)^M^ |
|  |  |  |  | B.C. Moon *2017-69-5* (KIOM)^M^ |
| 2 |  | Naju-si, Jeollanam-do, Korea | 35°02'12.6"N 126°42'03.4"E | S. Yang *2019-66-1* (KIOM)^C, M, B^ |
|  |  |  |  | S. Yang *2019-66-2* (KIOM)^M^ |
|  |  |  |  | S. Yang *2019-66-3* (KIOM)^M^ |
|  |  |  |  | S. Yang *2019-66-4* (KIOM)^M^ |
|  |  |  |  | S. Yang *2019-66-5* (KIOM)^M^ |
| 3 |  | Cheongju-si, Chungcheongbuk-do, Korea | 36°43'35.4"N 127°27'37.5"E | S. Yang *2016-26-1* (KIOM)^M, B^ |
|  |  |  |  | S. Yang *2016-26-2* (KIOM)^M^ |
|  |  |  |  | S. Yang *2016-26-3* (KIOM)^M^ |
|  |  |  |  | S. Yang *2016-26-4* (KIOM)^M^ |
|  |  |  |  | S. Yang *2016-26-5* (KIOM)^M^ |
| 4 |  | Sangju-si, Gyeongsangbuk-do, Korea | 36°32'19.6"N 128°01'06.9"E | B.C. Moon *2017-76-1* (KIOM)^M, B^ |
|  |  |  |  | B.C. Moon *2017-76-2* (KIOM)^M^ |
|  |  |  |  | B.C. Moon *2017-76-3* (KIOM)^M^ |
|  |  |  |  | B.C. Moon *2017-76-4* (KIOM)^M^ |
|  |  |  |  | B.C. Moon *2017-76-5* (KIOM)^M^ |
| 5 |  | Seongju-gun, Gyeongsangbuk-do, Korea | 36°29'23.3"N 128°22'09.4"E | B.C. Moon *2017-74-1* (KIOM)^M, B^ |
|  |  |  |  | B.C. Moon *2017-74-2* (KIOM)^M^ |
|  |  |  |  | B.C. Moon *2017-74-3* (KIOM)^M^ |
|  |  |  |  | B.C. Moon *2017-74-4* (KIOM)^M^ |
|  |  |  |  | B.C. Moon *2017-74-5* (KIOM)^M^ |
| 6 | *Trichosanthes kirilowii*  var. *japonica* | Sacheon-si,  Gyeongsangnam-do, Korea | 34°57'20.7"N 128°02'03.4"E | S. Yang *2017-18-1* (KIOM)^C, M, B^ |
|  |  |  |  | S. Yang *2017-18-2* (KIOM)^M^ |
|  |  |  |  | S. Yang *2017-18-3* (KIOM)^M^ |
|  |  |  |  | S. Yang *2017-18-4* (KIOM)^M^ |
|  |  |  |  | S. Yang *2017-18-5* (KIOM)^M^ |
| 7 |  | Seogwipo-si, Jeju-do, Korea | 33°18'50.5"N 126°49'43.5"E | S. Yang *2017-166-1* (KIOM)^C, M, B^ |
|  |  |  |  | S. Yang *2017-166-2* (KIOM)^M^ |
|  |  |  |  | S. Yang *2017-166-3* (KIOM)^M^ |
|  |  |  |  | S. Yang *2017-166-4* (KIOM)^M^ |
|  |  |  |  | S. Yang *2017-166-5* (KIOM)^M^ |
| 8 |  | Namhae-gun, Gyeongsangnam-do, Korea | 34°55'05.0"N 128°01'54.9"E | B.C. Moon *2016-253-1* (KIOM)^M, B^ |
|  |  |  |  | B.C. Moon *2016-253-2* (KIOM)^M^ |
|  |  |  |  | B.C. Moon *2016-253-3* (KIOM)^M^ |
|  |  |  |  | B.C. Moon *2016-253-4* (KIOM)^M^ |
|  |  |  |  | B.C. Moon *2016-253-5* (KIOM)^M^ |
| 9 |  | Sacheon-si, Gyeongsangnam-do, Korea | 34°57'20.7"N 128°02'03.4"E | B.C. Moon *2016-245-1* (KIOM)^M, B^ |
|  |  |  |  | B.C. Moon *2016-245-2* (KIOM)^M^ |
|  |  |  |  | B.C. Moon *2016-245-3* (KIOM)^M^ |
|  |  |  |  | B.C. Moon *2016-245-4* (KIOM)^M^ |
|  |  |  |  | B.C. Moon *2016-245-5* (KIOM)^M^ |
| 10 |  | Seogwipo, Jeju-do, Korea | 33°27'47.9"N 126°54'49.0"E | J.H. Song *2020-135-1* (KIOM)^M, B^ |
|  |  |  |  | J.H. Song *2020-135-2* (KIOM)^M^ |
|  |  |  |  | J.H. Song *2020-135-3* (KIOM)^M^ |
|  |  |  |  | J.H. Song *2020-135-4* (KIOM)^M^ |
|  |  |  |  | J.H. Song *2020-135-5* (KIOM)^M^ |
| 11 | *Trichosanthes rosthornii* | Qiubei, Wenshan,  Yunnan, China | 24°06'32.6"N 104°08'02.1"E | X. Zhou *n.s.* (KIOM)^C, M, B^ |

^C^, plastid genomic study; ^M^, morphology. ^B,^ ITS phylogenic tree analysis

**Table S2 Primers used for validation of junctions among single copy regions (SCs) and Inverted Repeat regions (IR)**

| Primer name | Primer sequence (5`>3`) | Position |
| --- | --- | --- |
| TriLSCF | GCGCTTTGTGAGGGGACATA | LSC_IRa |
| TriLSCR | GCGCTTTGTGAGGGGACATA |  |
| TriISF | CACTTCCGAAACGAAGGGGA | IRa_SSC |
| TriISR | AGCCGGCTTATTCATCTTTACA |  |
| TriSIF | CATCCCGAGCTAGAGTAAGCG | SSC_IRb |
| TriSIR | GGCAGAATACCGTCACCCAT |  |
| TriILF | AATCACACTTGGGAAGGGGG | IRb_LSC |
| TriILR | AGCTGCTGTTGAAGTTCCATCT |  |

**Table S3 PCR-based sequence validation of junctions among single copy regions (SCs) and Inverted Repeat regions (IR)**

| Species | location | PCR-based sequence (bp) | Plastid sequence (bp) | start | end | Identities | % |
| --- | --- | --- | --- | --- | --- | --- | --- |
| *T. kirilowii* var. *kirilowii* 1 | LSC_IRa | 699 | 156,797 | 85,624 | 86,322 | 100 |  |
|  | IRa_SSC | 736 |  | 111,934 | 112,669 | 100 |  |
|  | SSC_IRb | 739 |  | 130,276 | 131,014 | 100 |  |
|  | IRb_LSC | 774 |  | 156,338 | 313 | 100 |  |
| *T. kirilowii* var. *kirilowii 2* | LSC_IRa | 699 | 156,790 | 85,624 | 86,322 | 100 |  |
|  | IRa_SSC | 736 |  | 111,934 | 112,663 | 100 |  |
|  | SSC_IRb | 739 |  | 130,269 | 131,007 | 100 |  |
|  | IRb_LSC | 774 |  | 156,331 | 313 | 100 |  |
| *T. kirilowii* var. *japonica* 1 | LSC_IRa | 701 | 157,155 | 85,814 | 86,514 | 100 |  |
|  | IRa_SSC | 736 |  | 112,161 | 112,896 | 100 |  |
|  | SSC_IRb | 745 |  | 130,592 | 131,336 | 100 |  |
|  | IRb_LSC | 773 |  | 156,695 | 312 | 100 |  |
| *T. kirilowii* var. *japonica* 2 | LSC_IRa | 701 | 157,066 | 85,761 | 86,461 | 100 |  |
|  | IRa_SSC | 736 |  | 112,090 | 112,825 | 100 |  |
|  | SSC_IRb | 745 |  | 130,521 | 131,265 | 100 |  |
|  | IRb_LSC | 773 |  | 156,606 | 312 | 100 |  |
| *T. rosthornii* | LSC_IRa | 699 | 157,097 | 85,818 | 86,516 | 100 |  |
|  | IRa_SSC | 730 |  | 112,123 | 112,852 | 100 |  |
|  | SSC_IRb | 739 |  | 130,580 | 131,318 | 100 |  |
|  | IRb_LSC | 768 |  | 156,637 | 307 | 100 |  |

**Table S4 Plant samples used for validation of the new indel markers.**

| No. | Species | Collection information | Coordinates | Voucher number |
| --- | --- | --- | --- | --- |
| 1 | *T. kirilowii* var. *kirilowii* | Cheongju-si, Chungcheongbuk-do, Korea | 36°32'32.3"N 127°28'50.3"E | MBC_KIOM-2017-69 |
| 2 |  | Naju-si, Jeollanam-do, Korea | 35°02'12.6"N 126°42'03.4"E | YSG_KIOM-2019-66 |
| 3 |  | Hadong-gun, Gyeongsangnam-do, Korea | 35°01'55.0"N 127°51'05.0"E | KIOM201701018843 |
| 4 | *T. kirilowii* var*. japonica* | Sacheon-si, Gyeongsangnam-do, Korea | 35°02'12.6"N 126°42'03.4"E | MBC_KIOM-2017-55 |
| 5 |  | Tongyeong-si, Gyeongsangnam-do, Korea | 34°45'57.2"N 128°24'22.3"E | MBC_KIOM-2017-58 |
| 6 |  | Geoje-si, Gyeongsangnam-do, Korea | 34°46'20.6"N 128°38'16.6"E | MBC_KIOM-2017-63 |
| 7 | *T. rosthornii* | Wenshan, Yunnan, China | 34°57'20.7"N 128°02'03.4"E | KIOM201701018518 |
| 8 | *T. rubriflos* | Wenshan, Yunnan, China | 34°57'20.7"N 128°02'03.4"E | KIOM201701018768 |

**Table S5 Information regarding the *Trichosanthes* commercial products**

| No. | Crude drugs name | Tissue | Commercial product | Origin | Voucher NO. |
| --- | --- | --- | --- | --- | --- |
| 1 | Trichosanthis Fructus | Fruit | A company | Korea | 2-15-0508 |
| 2 |  |  | D company | Korea | 2-12-0154 |
| 3 |  |  | C company | Korea | 2-19-0362 |
| 4 | Trichosanthis Radix | Root | E company | Korea | 2-11-0140 |
| 5 |  |  | F company | Korea | 2-09-0030 |
| 6 |  |  | C company | Korea | 2-19-0364 |
| 7 |  |  | A company | China | 2-11-0152 |
| 8 |  |  | B company | China | 2-15-0175 |
| 9 |  |  | D company | Korea | 2-18-0061 |
| 10 | Trichosanthis Semen | Seed | D company | Korea | 2-17-0166 |
| 11 |  |  | A company | Korea | 2-09-0026 |
| 12 |  |  | A company | China | 2-10-0075 |
| 13 |  |  | B company | China | 2-15-0044 |
| 14 |  |  | G company | Korea | 2-16-0040 |
| 15 |  |  | H company | China | 2-16-0110 |

Table S6 Primer information for indel markers Tri, TRr and TRtt

| Primer name | Primer sequence (5`>3`) | Position |
| --- | --- | --- |
| Tri_F | GAGAAAAGCCGGCTATCGGA | *trnT-UGU-trnL-UAA* |
| Tri_R | TACCCTTAGGGCAGATCCTCT |  |
| TRr_F | TGAGGCATCCTAACAGACCG | *rrn4.5-rrn5* |
| TRr_R | GGCCCCAATTCTTGACTGGA |  |
| TRtt_F | AGATGTCCTGAACCACTAGA | *trnE-UUC-trnT-GGU* |
| TRtt_R | TTGTTTCATCATAAGACCGATCC |  |

**Table S7 Plastid genomes from NCBI used for phylogenetic analysis**

| No. | Taxon | GenBank accession number |
| --- | --- | --- |
| 1 | *Trichosanthes kirilowii* | MN542395 |
| 2 | *Trichosanthes kirilowii* | MT211646 |
| 3 | *Trichosanthes kirilowii* | MT211647 |
| 4 | *Trichosanthes baviensis* | NC_046864 |
| 5 | *Trichosanthes tricuspidata* | NC_046866 |
| 6 | *Trichosanthes tubiflora* | NC_046867 |
| 7 | *Trichosanthes homophylla* | NC_046868 |
| 8 | *Trichosanthes truncata* | NC_046875 |
| 9 | *Trichosanthes wallichiana* | NC_046882 |
| 10 | *Trichosanthes nervifolia* | NC_046883 |
| 11 | *Trichosanthes pilosa* | NC_046884 |
| 12 | *Trichosanthes lobata* | NC_046885 |
| 13 | *Cucumis melo* var. *makuwa* | MF536700 |
| 14 | *Cucumis melo* var. *momordica* | MF536701 |

**Table S8 Raw reads and trimmed reads data**

| **Scientific name** | **raw reads** | **Trimmed reads** | | **Total raw bases** | **Trimmed bases** | |
| --- | --- | --- | --- | --- | --- | --- |
| *T. kirilowii* var. *kirilowii* 1 | 5,107,006 | 4,381,209 | 85.79% | 1,537,208,806 | 1,157,756,715 | 75.32% |
| *T. kirilowii* var. *kirilowii* 2 | 6,230,038 | 5,388,318 | 86.49% | 1,875,241,438 | 1,502,308,792 | 80.11% |
| *T. kirilowii* var. *japonica* 1 | 5,760,168 | 4,959,166 | 86.09% | 1,727,549,308 | 1,201,963,309 | 69.58% |
| *T. kirilowii* var. *japonica* 2 | 5,343,386 | 4,618,215 | 86.43% | 1,602,100,858 | 1,122,618,478 | 70.07% |
| *T. rosthornii* | 6,190,270 | 5,383,287 | 86.96% | 1,855,651,181 | 1,316,515,437 | 70.95% |

Table S9 Genome assembly information for five *Trichosanthes* plastid genomes

| Scientific name | Aligned reads (#) | Coverage (x) | Plastid  genome length (bp) |
| --- | --- | --- | --- |
| *T. kirilowii* var. *kirilowii* 1 | 347,582 | 570.44 | 156,797 |
| *T. kirilowii* var. *kirilowii 2* | 307,368 | 546.97 | 156,790 |
| *T. kirilowii* var. *japonica* 1 | 559,386 | 851.56 | 157,155 |
| *T. kirilowii* var. *japonica* 2 | 199,436 | 303.12 | 157,066 |
| *T. rosthornii* | 55,359 | 83.73 | 157,097 |

Table S10 Genic introns in five *Trichosanthes* plastid genomes

| *T. kirilowii* var. *kirilowii* | Gene | region | exon I | intron I | exon II | intron II | exon III |
| --- | --- | --- | --- | --- | --- | --- | --- |
| 1 | *trnK-UUU* | LSC | 37 | 2501 (2507) * | 29 |  |  |
| 2 | *rps16* | LSC | 40 | 855 (839) | 215 |  |  |
| 3 | *trnG-UCC* | LSC | 21 | 688 (675) | 50 |  |  |
| 4 | *atpF* | LSC | 144 | 761 (757) | 411 |  |  |
| 5 | *rpoC1* | LSC | 435 | 755 | 1611 |  |  |
| 6 | *ycf3* | LSC | 126 | 729 | 228 | 740 (739) | 156 |
| 7 | *trnL-UAA* | LSC | 37 | 562 (564) | 50 |  |  |
| 8 | *trnV-UAC* | LSC | 38 | 605 | 37 |  |  |
| 9 | *rps12* | LSC | 232 |  | 26 |  | 114 |
| 10 | *clpP* | LSC | 69 | 818 | 294 | 617 | 228 |
| 11 | *petB* | LSC | 6 | 755 | 648 |  |  |
| 12 | *petD* | LSC | 9 | 728 | 474 |  |  |
| 13 | *rpl16* | LSC | 9 | 1101 | 399 |  |  |
| 14 | *rpl2* | LSC | 393 | 662 | 435 |  |  |
| 15 | *ndhB* | IR | 777 | 686 | 756 |  |  |
| 16 | *trnI-GAU* | IR | 56 | 937 | 37 |  |  |
| 17 | *trnA-UGC* | IR | 35 | 802 | 38 |  |  |
| 18 | *ndhA* | SSC | 552 | 1152 | 540 |  |  |
| *T. rosthornii* | Gene | region | exon I | intron I | exon II | intron II | exon III |
| 1 | *trnk-UUU* | LSC | 37 | 2507 | 29 |  |  |
| 2 | *rps16* | LSC | 40 | 839 | 215 |  |  |
| 3 | *trnG-UCC* | LSC | 21 | 688 | 50 |  |  |
| 4 | *atpF* | LSC | 144 | 758 | 411 |  |  |
| 5 | *rpoC1* | LSC | 435 | 764 | 1611 |  |  |
| 6 | *ycf3* | LSC | 126 | 722 | 228 | 740 | 156 |
| 7 | *trnL-UAA* | LSC | 37 | 514 | 50 |  |  |
| 8 | *trnV-UAC* | LSC | 38 | 605 | 37 |  |  |
| 9 | *rps12* | LSC | 232 |  | 26 |  | 114 |
| 10 | *clpP* | LSC | 69 | 820 | 294 | 630 | 228 |
| 11 | *petB* | LSC | 6 | 754 | 648 |  |  |
| 12 | *petD* | LSC | 9 | 728 | 474 |  |  |
| 13 | *rpl16* | LSC | 9 | 1105 | 399 |  |  |
| 14 | *rpl2* | LSC | 393 | 662 | 435 |  |  |
| 15 | *ndhB* | IR | 777 | 686 | 756 |  |  |
| 16 | *trnI-GAU* | IR | 56 | 941 | 37 |  |  |
| 17 | *trnA-UGC* | IR | 35 | 802 | 38 |  |  |
| 18 | *ndhA* | IR | 546 | 1153 | 540 |  |  |

*Parentheses indicate *Trichosanthes kirilowii* var*. japonica,* when different
